# Current status of Tele-speech language therapy by type and support for patients with post-stroke aphasia: A scoping review

**DOI:** 10.1371/journal.pone.0319805

**Published:** 2025-03-25

**Authors:** Yuhei Kodani, Shinsuke Nagami, Ayaka Yokozeki, Shinya Fukunaga, Katsuya Nakamura, Hikaru Nakamura

**Affiliations:** 1 Department of Speech and Hearing Therapy, Faculty of Rehabilitation Sciences, Kawasaki University of Health and Welfare, Kurashiki, Okayama, Japan; 2 Graduate School of Health and Welfare, Okayama Prefectural University, Soja, Okayama, Japan; 3 Department of Communication Disorders, School of Rehabilitation Sciences, Health Sciences University of Hokkaido, Ishikari-gun, Hokkaido, Japan; 4 Department of Neurosurgery, Takamatsu Red Cross Hospital, Takamatsu, Kagawa, Japan; Catalan Institute of Health: Institut Catala De La Salut, SPAIN

## Abstract

**Purpose:**

The purpose of this study was to classify and analyze trends in the assessment and training methods used in telepractice speech-language therapy (Tele-SLT) for people with aphasia (PWA), according to the type of Tele-SLT (synchronous, asynchronous, or combined). This study particularly aimed to identify gaps that prevent the establishment of Tele-SLT, a field that has gained significant attention post-COVID-19 pandemic.

**Design:**

A scoping review was conducted following the PRISMA-ScR guidelines.

**Setting and participants:**

Included were research articles on Tele-SLT for individuals aged 18 years or older diagnosed with post-stroke aphasia. Articles in both English and Japanese were reviewed, using five online databases (Medline, Embase, PsycInfo, Cochrane Library, and ICHUSHI Web).

**Methods:**

Studies involving Tele-SLT were categorized by support methods, content, study design, and outcomes. The quality of the extracted studies was also assessed. We also assessed the quality of the selected studies and performed a meta-analysis of some of the results.

**Results:**

Of the initial 1,484 articles, 35 met the eligibility criteria. Regarding Tele-SLT support methods, 3 articles (8.57%) focused on assessment methods, while 32 (91.43%) focused on training methods. Fourteen articles (40.00%) employed synchronous Tele-SLT delivery, 20 (57.14%) employed asynchronous delivery, and 1 (2.86%) employed a combined approach. The methodological quality of 27 (77.14%) of the included Tele-SLT articles was rated as ‘Low’. A meta-analysis of randomized controlled trials on Tele-SLT demonstrated that asynchronous training was effective for language function

**Conclusions and significance:**

This study highlights the need for more research, particularly on remote assessment and synchronous training methods, in Tele-SLT for PWA. Furthermore, this study emphasizes the need for improved research methodologies in this area. To provide high-quality support for PWA who have faced challenges accessing in-person speech-language therapy since the COVID-19 pandemic, further research and development of Tele-SLT implementation guidelines are needed

## Introduction

The incidence of stroke is increasing, especially among the elderly in an aging society [1,2]. As the number of stroke survivors increases, so too does the number of patients with aphasia (PWA), who face difficulties with language skills (listening, speaking, reading, and writing), communication (situational language use), well-being, and quality of life (QOL) [3–7]. Additionally, the COVID-19 epidemic has reduced PWA’s access to speech-language therapy (SLT) and limited opportunities for social interaction [8–10]. In this context, tele-lingual therapy (Tele-SLT) has become increasingly important for PWA [11].

Tele-SLT is a method of providing SLT using electronic devices and technology [12,13]. This Tele-SLT assistance can be divided into “assessment” (tele-assessment), which measures and quantitatively translates the patient’s condition, and “training” (tele-training), which aims to resolve the patient’s difficulties [14]. Furthermore, they can be provided in a synchronous manner, with a speech-language pathologist (SLP) providing SLT in real time via videoconferencing, or in an asynchronous manner, without the presence of an SLP and at one’s own pace, or combined types, in which a combination of both is provided [15–17]. The flexible combination of these methods according to the PWA’s needs allows for the provision of Tele-SLT tailored to individual requirements. Therefore, the demand for Tele-SLT has increased since the COVID-19 craze [18], but it has not yet reached the same level of diffusion as traditional methods [19,20].

Although the reasons for the struggle with Tele-SLT diffusion are not fully understood, a review of previous studies [15,21–23] suggests that one barrier may be the way information on Tele-SLT for PWA is organized and interpreted. Specifically, it is not well structured in terms of how it is provided and supports the PWA. In other words, there is a lack of clarity about what reliable assessment and training methods exist and how they can be delivered remotely for SLT for PWA. It is undeniable that the absence of reference standards for providing remote SLT has led to situations where it is needed but not provided.

Therefore, this study will use a scoping review approach to investigate Tele-SLT for post-stroke PWA, based on a framework of remote support methods: assessment methods, training methods, and delivery methods, including synchronous, asynchronous, and combined approaches. The significance of this study lies in comprehensively collecting, sorting, and disseminating information that will contribute to the dissemination of Tele-SLT and increase its provision to PWA who have experienced delayed access to professional support since the COVID-19 epidemic.

## Methods

### Study design

Our aim was to systematically identify and analyze the methods (assessment, training) and delivery (synchronous, asynchronous, combined) of Tele-SLT support for post-stroke PWA. Additionally, we sought to identify gaps in the current field of research. To achieve this objective, we used a scoping review methodology [24]. It is important to note that no published articles describe the protocol of this study.

### Information sources and evidence retrieval

This scoping review followed the guidelines of the Preferred Reporting Items for Systematic Reviews and Meta-Analyses extension for Scoping Reviews [25]. We achieved compliance with these guidelines [25]. Searches were conducted in the online databases of Medline [PubMed Interface], Embase, PsycInfo, Cochrane Library, and the ICHUSHI Web (Japan). The search strategy, designed by the expert medical secretary, is detailed in S1 Table. The first search was initiated on March 30, 2023, without specifying the year of publication. No manual search of the grey literature or reference lists of the included articles was performed.

### Selection criteria

We included studies that investigated the assessment or training methods of Tele-SLT for PWA following a stroke. Studies were excluded if they: 1) involved participants under 18 years of age; 2) combined Tele-SLT with in-person SLT; or 3) were review articles (e.g., systematic reviews, scoping reviews, narrative reviews), case reports, qualitative studies, cost-effectiveness analyses, books, conference proceedings, or study protocols. Full details of the inclusion and exclusion criteria are provided in Table 1.

**Table 1:** Inclusion and Exclusion Criteria.

Inclusion criteria	Exclusion criteria
Investigated Tele-SLT assessment or training methods for adults with PWA following a stroke. Included participants aged 18 years or older. Were published in English or Japanese.	Included any participants under 18 years of age, or any participants with traumatic brain injury, primary progressive aphasia, dementia, cancer, or conditions other than stroke. Provided in-person SLT as part of the training intervention, except in RCTs, quasi-RCTs where in-person SLT was provided to both the Tele-SLT intervention and control groups. Were published in a language other than English or Japanese. Were review articles (e.g., systematic reviews, meta-analyses, scoping reviews, narrative reviews), case reports, study protocols, qualitative studies, cost-effectiveness analyses, books, or conference proceedings

Abbreviations: *Tele-SLT* telepractice speech-language therapy, *PWA* people with aphasia, *SLT* speech-language therapy, *RCT* Randomized Controlled Trial

### Screening method

Two authors (YK and SN) independently screened titles and abstracts of identified articles. Subsequently, two other authors (YK and AY) independently reviewed the full texts of the selected articles for inclusion. Any discrepancies between authors during the screening process were resolved through discussion. If a consensus could not be reached, a third author (HN) made the final decision.

### Data extraction process

Two authors (YK and AY) independently reviewed the full texts of articles selected in the second screening and extracted data. Discrepancies in data extraction were resolved through discussion between the two authors. If consensus could not be reached, a third author (HN) made the final decision. For studies on tele-assessment methods, the following information was collected: author, year of publication, country of the first author, participant characteristics (e.g., sex, age), number of participants, domains covered (e.g., language function, communication, well-being, QOL), software used, electronic devices used, and scale accuracy (e.g., reliability, validity). For studies on tele-training methods, the following information was collected: author, year of publication, study design, participant characteristics (sex, age), number of participants, domains covered (language function, communication, well-being, QOL), software used, electronic devices used, and training outcomes. One author (YK) managed the data using Rayyan reference management software and compiled the data into tables using Excel. Any missing articles were obtained by contacting the corresponding authors.

### Study quality assessment

Two authors (YK, AY) independently assessed the quality of the studies, with the third author (SF) resolving any disagreements. The evaluation checklist [21,26,27], partially adapted by the authors to fit this study’s inclusion criteria, consisted of five categories: study design, sample size, demographic variables, aphasia variables (including type classification, severity, and duration since onset), telemedicine characteristics, and data collection methods (specifically, the use of standardized rating scales). Each category was assigned a rating of high, medium, or low (S2 Table). A study was considered to be of high quality if it received no low” ratings and at least four “high” ratings. If a study has no “low” ratings but does not meet the criteria for high quality, it is classified as “medium.” Otherwise, it is rated as “low.”

### Meta-analysis of the training effects of Tele-SLT

A meta-analysis was conducted using Review Manager 5.4 to evaluate the effects of Tele-SLT training reported in randomized controlled trials (RCTs). The analysis focused on language and communication outcomes. Mean differences (MD), standardized mean differences (SMD) and 95% confidence intervals (CI) were calculated for each outcome using extracted means and standard deviations. Heterogeneity was assessed using I^2^. A fixed-effects model was used when heterogeneity was not significant (*P* >  0.01), and a random-effects model was used when heterogeneity was significant (*P* ≤  0.01). Outcome measures included, for language function, the Aphasia Quotient (AQ) and its subscales (spontaneous speech, comprehension, naming and repetition) of the Western Aphasia Battery (WAB), WAB-Revised (WAB-R), the Aachen Aphasia Test, and the Boston Naming Test (BNT). For communication function, outcome measures included the Communication Activities Log (CAL), the Communication Effectiveness Index (CETI), and the Communication Abilities in Daily Living (CADL). SMD were calculated when analyzing different outcomes within the same model, whereas MD were calculated for homogenous outcomes. These procedures were based on previous meta-analyses regarding aphasia treatment [28,29].

## Results

### Search results

**Fig 1:**
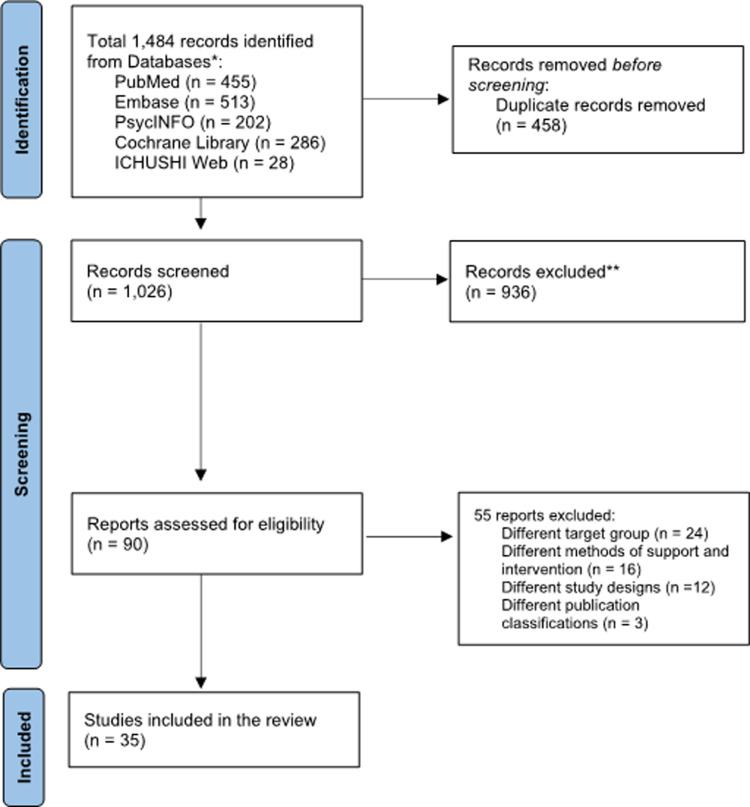
PRISMA Flow Diagram Illustrating the Selection of Articles Included in the Scoping Review.

The initial search yielded a total of 1,484 articles. After removing duplicates, 1,026 articles underwent primary screening based on title and abstract. This screening narrowed the list to 90 articles, which underwent a secondary comprehensive full-text review. Finally, 35 articles met the inclusion criteria and were included in the current scoping review (Fig 1). S3 Table presents the list of articles included in the secondary screening, the evaluators’ (YK, AY) judgments for each, the final decisions, and the reasons for exclusion.

### Trends in selected articles

#### Overall trend.

**Fig 2:**
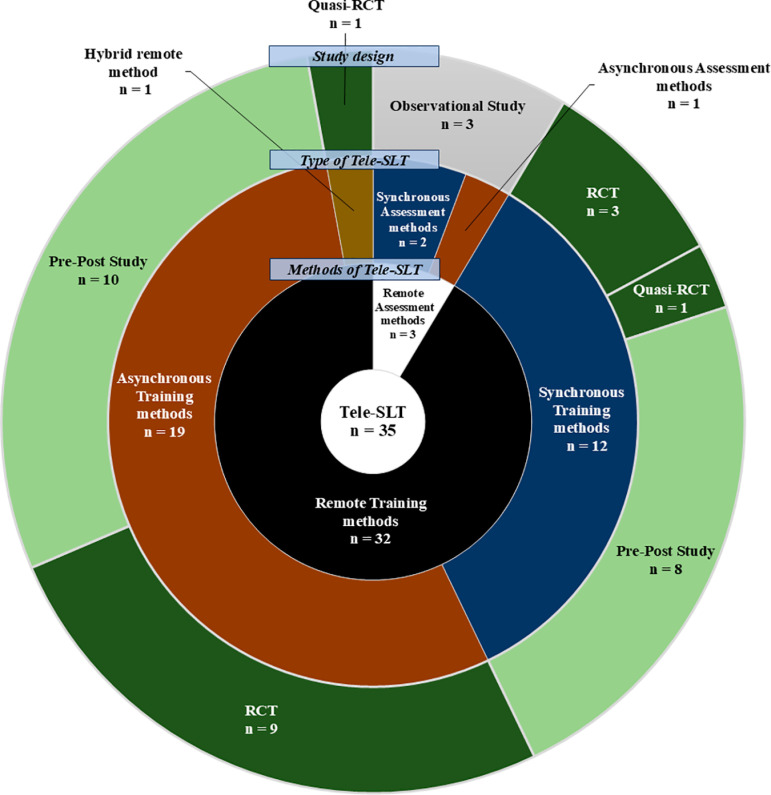
How to Support and Deliver Tele-SLT and the Design of Studies.

Regarding Tele-SLT support methods, three articles (8.57%) focused on assessment, while 32 (91.43%) focused on training. Regarding delivery methods, 14 articles (40.00%) used synchronous Tele-SLT, 20 (57.14%) used asynchronous Tele-SLT, and 1 (2.86%) used a combined approach. Across all 35 articles, 859 individuals with PWA participated, of whom 693 individuals with chronic PWA were included in 31 articles (88.57%). Study designs for assessment methods were all cross-sectional (n =  3/3). For training methods, designs included pre-post studies (n =  18/32, 56.25%), quasi-randomized controlled trials (quasi-RCTs) (n =  2/32, 6.25%), and RCTs (n =  12/32, 37.50%). All articles were published in English. Tele-SLT support methods, delivery methods, and study designs are summarized in Fig 2.

#### Trends in research on synchronous Tele-SLT.

Fourteen studies investigated synchronous Tele-SLT, comprising two studies on assessment methods and twelve on training methods (Tables 2 and 3). The assessment studies focused on measuring language function and QOL [30,31]. Training studies targeted language function (n =  8) [32–34,36,39–42], communication (n =  8) [34,39,40,35,37,38,42,43], QOL (n =  7) [34,36,40,37,38,43], and well-being (n =  3) [34,40,43]. Seven studies utilized software specifically designed for PWA. Access2Aphasia was used for assessment [30], while EVA Park [34], Oralys Tele Therapy [35], The Web-based dual card game [36], Rehabilitation Gaming System for aphasia [39,41], and NeuroVR 2.0 [40] were used for training.

**Table 2:** Details Regarding the Studies on Synchronous Assessment.

Author (year, country)	Participants	Number of participants	Sex of participants	Mean age of participants (SD)	Assessment area	Software	Machinery and tools	Results: reliability and validity
Guo Y.E. et al [30]. (2017, Singapore)	Chronic PWA	30	M23, F7	61.5 (12.03)	Language function, QOL	Access2Aphasia (software for remote evaluation of ALA and PALPA)	iPad	High correlations with the face-to-face condition, excellent intra-rater and inter-rater reliability were calculated.
Altaib, M. K., et al. [31] (2023, Saudi Araibia)	Chronic PWA	19	M16, F3	48.48 (11.57)	Language function	WebEx (video conferencing system software)	MacBook	There was no significant difference between the results of the Short Aphasia Test for Gulf Arabic speakers when administered remotely and when administered face-to-face, indicating criterion-related validity.

Abbreviations: *ALA* Assessment for Living with Aphasia, *PALPA* Psycholinguistic Assessments of Language Processing in Aphasia.

Note: We selected “domain of intervention” from “language function,” “communication,” “well-being,” and “quality of life” based on Wallace, S. J., et al. [5].

**Table 3:** Details Regarding the Studies on Synchronous Training.

Study design and basic attributes								
Author (year, country)	Study design	Participants	Number of participants in the intervention group	Number of participants in the control group	Sex of the intervention group	Sex of the control group	Mean age of the intervention group (SD)	Mean age of the control group (SD)
Agostini, M., et al. [32] (2014, Italy)	Pre-Post study	Chronic PWA	5	–	M4, F1	–	65.4 (5.7)	–
Furnas, D. W., et al. [33] (2014, USA)	Pre-Post study	Chronic PWA	2	–	M2	–	–	–
Marshall J. et al. [34] (2016, UK)	Quasi-RCT	Chronic PWA	10	10	M6, F4	–	59.0 (13.61)	56.6 (9.73)
Macoir J. et al. [35] (2017, Canada)	Pre-Post study	Chronic PWA	20	–	M14, F6	–	63.7 (10.1)	–
Pitt, R., et al. [36] (2017, Australia)	Pre-Post study	Chronic PWA	2	–	M1, F1	–	59.50 (26.16)	–
Pitt, R., et al. [37] (2019, Australia)	Pre-Post study	Chronic PWA	4	–	M2, F2	–	57.50 (16.18)	–
Pitt, R., et al. [38] (2019, Australia)	Pre-Post study	Chronic PWA	18	–	M9, F9	–	58.0 (14.87)	–
Grechuta, K., et al. [39] (2019, Spain)	RCT	Chronic PWA	9	8	M4, F5	M5, F3	55.67 (8.4)	53.50 (11.33)
Giachero A. et al. [40] (2020, Italy)	RCT	Chronic PWA	18	18	–	–	–	–
Grechuta, K., et al. [41](2020, Spain)	Pre-Post study	Chronic PWA	10	–	M5, F5	–	57.6 (9.9)	–
Øra, H. P., et al. [42] (2020, Norway)	RCT	Convalescent PWA and Chronic PWA	32	30	M19, F13	M22, F8	64.7 (11.7)	65.0 (12.2)
Cruis, M. et al. [43] (2021, UK)	Pre-Post study	Chronic PWA	29	–	M20, F9	–	61.3 (11.1)	–
**Intervention details**								
**Author (year, country)**	**Intervention area**	**Software**	**Machinery and tools**	**Intervention group training content**	**Control group training content**	**Frequency of training**	**Post-therapy results**	**Maintenance**
Agostini, M., et al. [32] (2014, Italy)	Language function	Skype	Laptop, webcam, headphones	Online picture naming task	Face-to-face naming task for picture words	8days, 8session	There was a significant improvement in the accuracy of naming words that had been trained in the same way as in person	Three weeks after training, naming test results were maintained.
Furnas, D. W., et al. [33] (2014, USA)	Language function	Adobe® Connect, software created independently (software that provides aphasia patients with vocabulary search training)	Desktop computer or laptop	Performed listening comprehension, speech, and typing tasks	–	8weeks, 3session/week(2hours/session)	Two participants showed improvements in their scores for the items on word production, sentence production, and WAB writing, and one participant showed an improvement in their WAB-AQ score.	After 12 weeks of training, the accuracy of naming words was maintained
Marshall J. et al. [34] (2016, UK)	Language function, Communication, Well-being, QOL	EVA Park (VR language training software)	Laptop	Individualized language and communication training, Conversation training	No treatment	5 weeks, 1 h daily	Significant improvement in CADL-2 and CCRSA	Improvement in CADL-2 maintained at 13-week follow-up
Macoir J. et al. [35] (2017, Canada)	Communication	Oralys TeleTherapy (software specialized for PACE)	Desktop computer	PACE training with 30 individually selected words	–	3 weeks, 3 sessions/week	Significant improvement in communication effectiveness scores, significant reduction in communication exchange time, significant reduction in the number of communication acts, and significant increase in communication diversity	Improvement in all outcome measures maintained at 6-week follow-up of training
Pitt, R., et al [36]. (2017, Australia)	Language function, QOL	The Web-based dual card game, Adobe connect	Desktop computer, webcam, headset microphone	Intensive Constraint-Induced Aphasia Training Implemented training based on the principle of naming	–	2weeks, 5sessions/week (3hours/session)	The CAT and ALA scores improved.	–
Pitt, R., et al. [37] (2019, Australia)	Communication, QOL	Adobe connect	Desktop computer or laptop, webcam, headset microphone	Communication in groups of 2-4 people	–	12 weeks, 1session/week (1.5 hours/session)	The CAT and ALA scores improved significantly.	–
Pitt, R., et al. [38] (2019, Australia)	Communication, QOL	Adobe connect	Desktop computer or laptop, webcam, headset microphone	Communication in groups of 2-4 people	–	12weeks	The ALA, the Quality of Communication Life Scale, the Communication Activity Checklist, and CAT scores significantly improved.	–
Grechuta, K., et al. [39] (2019, Spain)	Language function, Communication	Rehabilitation Gaming System for aphasia (software that provides training based on intensive language and movement therapy using virtual reality)	Desktop computer; motion-tracking sensor	PWA members conduct intensive language training and name training in a VR space	Standardized training for specific language disorders	8weeks, 5sessions/week (30-40 minutes/session)	The BDAE scores improved significantly within the treatment group, and the CAL scores improved significantly compared to the control group	After 8 weeks of training, it was confirmed that the results of the BDAE and CAL were maintained
Giachero A. et al. [40] (2020, Italy)	Language function, Communication, Well-being, QOL	NeuroVR 2.0 (Software for language and cognitive function training using VR)	Projector, Desktop computer or laptop	Conversation and language function training based on virtual scenarios in VR	Face-to-face conversation training	24 weeks, 2 sessions/week (2 h/session)	Significant improvement in AAT, C.A.P.P.A. WHOQoL Questionnaire, VASES	–
Grechuta, K., et al. [41] (2020, Spain)	Language function	Rehabilitation Gaming System for aphasia (software that provides training based on intensive language and movement therapy using virtual reality)	Desktop computer, VR headset, motion -tracking sensor	PWA members conduct intensive language training and name training in a VR space	–	8weeks, 5sessions/week (30-40 minutes/session)	The Vocabulary Test scores significantly improved during and after training	After 8 weeks of training, the results of The Vocabulary Test were significantly improved compared to before training
Øra, H. P., et al. [42] (2020, Norway)	Language function, Communication	Cisco Jabber/Acano (video conferencing software)	Laptop, webcam, external speakers	Practice word naming, reading comprehension, and communication tailored to individual needs	Standard Care	8weeks, 5days/week(60minutes/session)	The scores on the sub-tests of the Norwegian Basic Aphasia Assessment and the CETI improved significantly	After 12 weeks of training, the results of the subtests of the Norwegian Basic Aphasia Assessment improved compared to the control group, while the results of the CETI test were maintained
Cruis, M. et al. [43] (2021, UK)	Communication, QOL, Well-being	Skype	Desktop computer or laptop, iPad	Instruction on the use of equipment and conversation training, as well as practice of communication skills in this context	–	8 weeks, twice a week (1 h/session)	Social network assessment showed an increase in the number of social contacts and a significant improvement in CCRSA and ALA	Social network coverage, CCRSA, and ALA maintained at 8-week follow-up

Abbreviations: *WAB* the Western Aphasia Battery, *WAB-AQ* the Western Aphasia Battery Aphasia Quotient*, CADL-2* Communication ADL Test-2, *CCRSA* Communication Confidence Rating Scale for Aphasia, *PACE* Patient-Accessible Communication Environment, *CAT* Comprehensive Aphasia Test, *ALA* Assessment for Living with Aphasia*, BDAE* Boston Diagnostic Aphasia Examination, *CAL* Communicative Activity Log, *AAT* Aachen Aphasia Test, *C.A.P.P.A* Conversation Analysis Profile For People with Aphasia, *WHOQOL* The World Health Organization Quality of Life, *VASES* Visual Analog Self-Esteem Scale, *CETI* Communicative Effectiveness Index.

Note: We selected “domain of intervention” from “language function,” “communication,” “well-being,” and “quality of life” based on Wallace, S. J., et al. [5].

Assessment methods were based on the Assessment of Living with Aphasia, the Psycholinguistic Assessments of Language Processing in Aphasia, and the Short Aphasia Test for Gulf Arabic speakers. The psychometric properties examined included intra-rater and inter-rater reliability, criterion validity, and construct validity. Training methods were based on Promoting Aphasics’ Communicative Effectiveness [35], Intensive Aphasia Therapy [36,39,41], and conversational training [37,38,40,42,43], with treatment durations ranging from eight days to 24 weeks. Eight studies reported on the maintenance of treatment effects at follow-up periods of 3 to 12 weeks [32–35,39,41–43].

#### Trends in research on asynchronous Tele-SLT.

Twenty studies investigated asynchronous Tele-SLT, including one study on assessment methods and nineteen on training methods (Tables 4 and 5). The assessment study focused on measuring language function (n =  1) [44]. Training studies focused on language function (n =  17) [45,46,48–56,58–63], communication (n =  7) [46–48,56,57,59,60], and improving QOL (n =  3) [58,60,62], as well as well-being (n =  1) [58]. Seventeen studies used software specifically designed for PWA. The Mobile Aphasia Screening Test was used for assessment [44]. Training programs utilized various software including Aphasia Scripts [46,47,57,63], Aphasia Mate [48], Oral Reading for Language in Aphasia [49,61], AphasiaRx [51,53], StepByStep aphasia software [50,59], and iAphasia [54].

**Table 4:** Details of the Studies of Asynchronous Assessment.

Author (year, country)	Participants	Number of participants	Sex of participants	Mean age of participants (SD)	Assessment area	Software	Machinery and tools	Results: reliability and validity
Choi, Y. H. et al. [44] (2015, Korea)	Chronic PWA	30	M25, F5	53.67 (5.27)	Language function	MAST (software version of FAST)	iPad	Inter-rater reliability, internal consistency reliability, and convergent validity (correlation with K-FAST and K-WAB-AQ) were good. Sensitivity and specificity for determining the presence of aphasia were also good.

Abbreviations: *K- WAB-AQ* Korean version the Western Aphasia Battery Aphasia Quotient, *MAST* Mobile Aphasia Screening Test, *K-FAST* Korean version Frenchay Aphasia Screening Test.

Note: We selected “domain of intervention” from “language function,” “communication,” “well-being,” and “quality of life” based on Wallace, S. J., et al. [5].

**Table 5:** Details Regarding the Studies on Asynchronous Training.

Study design and basic attributes								
Author (year, country)	Study design	Participants	Number of participants in the intervention group	Number of participants in the control group	Sex of the intervention group	Sex of the control group	Mean age of the intervention group (SD)	Mean age of the control group (SD)
Kats, R. C. et al. [45] (1997, USA)	RCT	Chronic PWA	21	34 (dummy stimulus 19, no treatment 15)	–	–	61.6 (10.0)	Dummy stimulus: 66.4 (6.0), No treatment: 62.8 (5.1)
Cherney, L. R., et al. [46] (2008, USA)	Pre-Post study	Chronic PWA	3	–	M2, F1	–	64.0 (12.77)	–
Manheim, L.M. et al. [47] (2009, USA)	Pre-Post study	Chronic PWA	20	–	M13, F7	–	54.80 (15.25)	–
Archibald, L. M. D., et al. [48] (2009, Canada)	Pre-Post study	Chronic PWA	8	–	M6, F2	–	71.13(11.12)	–
Cherney, L. R [49]. (2010, USA)	RCT	Chronic PWA	11	14	M 8, F 3	M8, F6	56.6 (9.2)	61.1 (14.8)
Palmer, R., et al. [50] (2012, UK)	RCT	Chronic PWA	16	17	M9, F7	M12, F5	69.5 (12.2)	66.2 (12.3)
van Vuuren, S., et al. [51] (2014, USA)	Pre-Post study	Chronic PWA	8	–	–	–	52.0 (14.0)	–
Kurland, J., et al. [52] (2014, USA)	Pre-Post study	Chronic PWA	5	–	M2, F3	–	67.6 (8.26)	–
Cherney, L.R., et al. [53] (2015, USA)	Pre-Post study	Chronic PWA	8	–	M6, F2	–	52.0 (14.0)	–
Choi, Y. H., et al. [54] (2016, Korea)	Pre-Post study	Chronic PWA	8	–	M4, F4	–	50.75 (8.88)	–
Kurland, J. et al. [55] (2018, USA)	Pre-Post study	Chronic PWA	21	–	M13, F8	–	66.4 (8.4)	–
Zhou, Q., et al. [56] (2018, China)	RCT	Convalescent PWA	10	10	M7, F3	M6, F4	59.80 (11.26)	56.50 (14.34)
Cherney, L.R. et al. [57] (2019, USA)	Pre-Post study	Chronic PWA	20	–	M14, F6	–	56.9 (8.4)	–
Maresca, G. et al. [58] (2019, Italy)	RCT	Convalescent PWA	15	15	M7, F8	M7, F8	51.1 (10.4)	51.4 (12.7)
Palmer, R., et al. [59] (2019, UK)	RCT	Chronic PWA	83	86	M47, F36	M54, F32	64.9 (13.0)	Control A: 64.9 (13.0), Control B: 63.8 (13.1)
Spaccavento, S. et al. [60] (2021, Italy)	RCT	Acute PWA	13	9	M9, F4	M7, F2	57.38 (9.23)	64.11 (15.04)
Cherney, L.R. et al. [61] (2021, USA)	RCT	Chronic PWA	19	13	M10, F9	M9, F4	58.27 (13.55)	55.19 (11.46)
Braley M., et al. [62] (2021, Italy)	RCT	Convalescent PWA	17	15	M10, F7	M8, F7	59.8 (10.0)	64.2 (9.9)
Cherney, L.R., et al. [63] (2022, USA)	Pre-Post study	Chronic PWA	16	–	M10, F6	–	61.54 (12.96)	–
**Intervention details**								
**Author (year, country)**	**Intervention area**	**Software**	**Machinery and tools**	**Intervention group training content**	**Control group training content**	**Frequency of training**	**Post-therapy results**	**Maintenance**
Kats, R. C. et al. [45] (1997, USA)	Language function	Digital program for visual matching and reading (developed for research)	Desktop personal computer	Word and Sentence Reading Assignment	Non-verbal, cognitive rehabilitation software, computer games, no treatment	3h per week for 26 weeks	PICA and WAB improved significantly	–
Cherney, L. R., et al. [46] (2008, USA)	Language function, Communication	AphasiaScripts^TM^ (software that uses animated agents that function as virtual therapists to support skript traning)	Desktop computer or laptop	Script training with a virtual therapist	–	9 weeks, 7 sessions/week (30minetes/session)	Two out of three participants showed improvements in content, grammar, and word production speed in two of the three scripts. One participant showed improvements in WAB-AQ and CETI scores	After 6 weeks of training, the WAB-AQ and CETI scores improved and were maintained
Manheim, L.M. et al. [47] (2009, USA)	Communication	Aphasia Scripts^TM^ (computer-based script training program)	Laptop	Script training with a virtual therapist	–	9 weeks, 30 min daily	BOSS-CD decreased (decreased sense of communication difficulty)	BOSS-CD values were maintained at the 6-week follow-up
Archibald, L. M. D., et al. [48] (2009, Canada)	Language function, Communication	AphasiaMate	Desktop computer or laptop	Language function tasks such as auditory comprehension, reading comprehension, meaning therapy, and calculation	–	15 weeks, at least once a week (60 minutes or more per session)	Significant improvement in the results of the lower-level tests for WAB and ASHA-FACS	–
Cherney, L. R [49]. (2010, USA)	Language function	Oral Reading for Language in Aphasia (ORLA)	Desktop computer or laptop	Comprehension of sentences by software, recitation of words, training on reading words aloud	Sentence comprehension, word recitation, word reading practice	6–22 weeks, 1–4 sessions/week (1 h/session)	More patients in the treatment group had improved WAB-AQ, and no significant difference in WAB-AQ change was noted between the treatment and control groups.	–
Palmer, R., et al. [50] (2012, UK)	Language function	The StepByStep aphasia software (software for aphasia training)	Desktop computer or laptop	Word Search Task	Participation in communication support groups, and everyday activities such as conversation, reading and writing	20 weeks, 3 sessions/week (20minutes/session)	The accuracy of word naming in the Object and Action Naming Battery has significantly improved	After 12 weeks of training, the accuracy of naming lifesaving words is maintained
van Vuuren, S., et al. [51] (2014, USA)	Language function	AphasiaRx™ (Software for language function training using script training methods)	Laptop	Script training with a virtual therapist	–	3 weeks, 6 sessions/week (up to 90minutes/session)	The accuracy of the words used in the 10 sentences practiced improved significantly compared to before training	After 3, 6, and 12 weeks of training, the accuracy of naming words in the trained script is maintained
Kurland, J., et al. [52] (2014, USA)	Language function	iBooks (with a playback function for videos such as audio comprehension of words edited in imove and pronunciation hints)	iPad	Vocabulary reading comprehension, repetition, and naming practice	–	24 weeks, 5-6 sessions/week(20minutes/session)	4-5 participants improve the accuracy of the expression of not only trained words but also untrained words	–
Cherney, L.R., et al. [53] (2015, USA)	Language function	AphasiaRx™ (Software for language function training using script training methods)	Laptop	Script training with a virtual therapist	–	3 weeks, 6 sessions/week (90 minutes/session)	Significant improvement in the accuracy of word naming in scripts	–
Choi, Y. H., et al. [54] (2016, Korea)	Language function	iAphasia (language training software)	iPad	Training tasks for auditory comprehension, reading comprehension, repetition, memorization, writing, and language fluency	–	4 weeks	Significant improvement in the lower ratings of WAB-AQ and WAB	Improvement in WAB-AQ maintained after 4 weeks of training
Kurland, J. et al. [55] (2018, USA)	Language function	iBooks	iPad	Training in calling pictures (objects and actions) presented in software	–	5–6 sessions a week for 24 weeks (20 min/session)	Significant improvements in calling performance for used and unused words during training	Maintain nominal performance at 16 weeks follow-up
Zhou, Q., et al. [56] (2018, China)	Language function, Communication	Language training software for people with aphasia from The Wispirit Inc. (66nao.com)	Desktop computer or laptop or Tablet device	Listening comprehension, reading comprehension, repetition, dictation, and writing practice exercises	Communication about family topics	4 weeks, 7 sessions/week (30 minutes/session)	Significant improvement in WAB and CADL scores	–
Cherney, L.R. et al. [57] (2019, USA)	Communication	Aphasia Scripts -VR	Desktop computer or laptop	Script training with a virtual therapist	–	1 time only, 60 min.	Training scripts were evaluated by NORLA-6 and showed significant improvement	These improvements were maintained for after 2weeks.
Maresca, G. et al. [58] (2019, Italy)	Language function, Well-being, QOL	Virtual Reality Rehabilitation System (VRRS-Tablet)	Tablet device	Object nomenclature training, sentence construction, sentence composition nomenclature training	Traditional speech therapy	12 weeks, 5 sessions/week (50 min/session)	Significant improvements in TT, ADRS, ENPA EQ-5D, and PIADS	Improvement was still observed in all outcomes after 12 weeks of training
Palmer, R., et al. [59] (2019, UK)	Language function, Communication	The StepByStep aphasia software (software for aphasia training)	Desktop computer or laptop or Tablet device	Search task for words related to PWA	Paper-based cognitive function activities (Sudoku, Spot the Difference, etc.)	24 weeks, 7 sessions/week (20–30 minutes/session)	Significant improvement in the results of the 100-word picture naming test	After 12 and 24 weeks of training, the results for word recognition were maintained
Spaccavento, S. et al. [60] (2021, Italy)	Language function, Communication, QOL	Software programs edited by Erickson (Edizioni Centro Studi Erickson, S.p.A.) for rehabilitation language skills training	Desktop computer or laptop	Software training for word and sentence expression and language comprehension	Therapist training in word and sentence expression and language comprehension	8 weeks, 5 sessions/week (50 min/session)	Significant improvement in AAT, FOQ-A, FAM, and QLQA	–
Cherney, L.R. et al. [61] (2021, USA)	Language function	Web-based Oral Reading for Language in Aphasia (ORLA® Web version)	Laptop, audio headset, and webcam	Listening comprehension, reading comprehension, recitation, and oral reading tasks using a virtual avatar of the therapist	PopCap’s commercial game Bejeweled 2©, which does not target language skills	6 weeks, 6 sessions/week (90 min/session)	Significant improvement in WAB-R-LQ	Further improvement was observed at the 6-week follow-up
Braley M., et al. [62] (2021, Italy)	Language function, QOL	Constant Therapy-Research (software that provides systematic and structured therapy like that usually provided by speech-language-hearing therapists)	Tablet device	Training that spans the cognitive, speech, and language domains	Independent practice using a aphasia treatment workbook	8 weeks, 5 sessions/week (30 minutes/session)	Significant improvement in WAB-AQ	–
Cherney, L.R., et al. [63] (2022, USA)	Language function	AphasiaScripts™ (software program that uses animated agents to function as virtual therapists to support aphasia patients’ speech training)	Laptop	Script training with a virtual therapist	–	3 weeks, 6 sessions/week (30 minutes/session)	Significant increase in the accuracy of words in the trained script and the number of words per minute	–

Abbreviations: *PICA* Porch Index of Communicative Ability, *WAB* the Western Aphasia Battery, *WAB-AQ* the Western Aphasia Battery Aphasia Quotient, *CETI* Communicative Effectiveness Index, *BOSS* Burden of Stroke Scale, *CD* Communication Difficulty, *ASHA-FACS* American Speech-Language-Hearing Association Functional Assessment of Communication Skills for Adults, *CADL* Communication ADL Test, *NORLA-6* Naming and Oral Reading for Language in Aphasia 6-Point Scale, *TT* Token Test, ADRS Aphasic Depression Rating Scale, ENPA Esame Neurologico Per l’Afasia, EQ-5D Euro-Qol-5D, *PIADS* Psychosocial Impact of Assistive Devices Scale, *AAT* Aachen Aphasia Test, *I-FOQ-A* Italian Version of Functional Outcome Questionnaire for Aphasia, *FAM* Functional Assessment Measure, *QLQA* Quality of Life Questionnaire for Aphasics, *WAB-R* -*LQ* Western Aphasia Battery-Revised Language Quotient.

Note: We selected “domain of intervention” from “language function,” “communication,” “well-being,” and “quality of life” based on Wallace, S.J., et al. [5].

The assessment study was based on the Korean version of the Frenchay Aphasia Screening Test and examined internal consistency, inter-rater reliability, criterion validity, and construct validity [44]. Training studies employed methods such as word comprehension and production training [45,48–50,52,54–56,58–62] and script training [46,47,51,53,57,63], with intervention durations ranged from single sessions to 26 weeks. Ten studies reported on the maintenance of treatment effects at follow-up periods of 2 to 24 weeks [46,47,50,51,54,55,57–59,61].

#### Trends in research on combined Tele-SLT.

The one combined study was a training method study (Table 6). This study focused on improving language function (n =  1) [64] by providing self-designed simultaneous and non-simultaneous training in Face Time and Power Point [64]. Results showed a significant improvement in performance on a unique calling task and confirmed maintenance of calling ability after 6 weeks of training.

**Table 6:** Details Regarding the Study on Combined Training.

Study design and basic attributes								
Author (year, country)	Study design	Participants	Number of participants in the intervention group	Number of participants in the control group	Sex of the intervention group	Sex of the control group	Mean age of the intervention group (SD)	Mean age of the control group (SD)
Woolf, C., et al. [64] (2016, UK)	Quasi-RCT	Chronic PWA	10	10	Remote from university: M 3, F 2, Remote from clinical site: M 4, F 1	Face-to-face: M 3, F 2, Attention control: M 4, F 1	Remote from university: 67.2 (6.98), Remote from clinical site: 58.6 (14.38)	Face-to-face: 57.8 (15.14), Attention control: 53.0 (13.93)
Intervention details								
Author (year, country)	**Intervention area**	**Software**	**Machinery and tools**	**Intervention group training content**	**Control group training content**	**Frequency of training**	**Post-therapy results**	**Maintenance**
Woolf, C., et al. [64] (2016, UK)	Language function	FaceTime, Power Point	iPad	Training in naming and understanding words, both synchronous and asynchronous	Comprehension, call training, simultaneous conversation training	4 weeks, twice a week (1 h/session)	Significant improvements in name calling performance for 100 items	Nominal results maintained at the 6-week follow-up

Note: We selected “domain of intervention” from “language function,” “communication,” “well-being,” and “quality of life” based on Wallace, S.J., et al. [5].

### The quality of Tele-SLT studies

**Fig 3:**
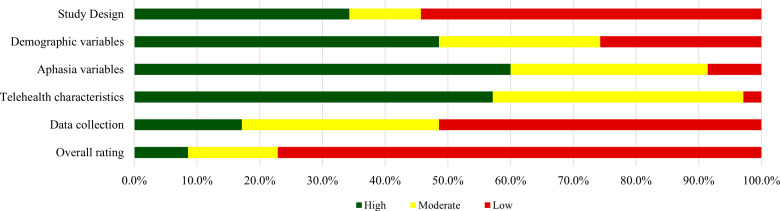
Summarize the Results of the Study Quality Assessment.

Two authors (YK and AY) demonstrated high agreement (97.14%, 34/35) in their assessments of the included studies. Three studies (8.57%) [40,42,62] received a rating of “High,” including both synchronous and asynchronous Tele-SLT training interventions. Five studies (14.29%) [31,34,39,59,60] were rated “Moderate,” encompassing synchronous Tele-SLT assessment and training, as well as asynchronous Tele-SLT training. The remaining 27 studies (77.14%) were rated as “Low” (Table 7). Notably, “Study Design” (lack of a control group or insufficient sample size in RCTs) and “Data Collection” (insufficient patient outcome measures) were the most frequent reasons for a “Low” rating (n =  19, 18; 54.29%, 51.43%) (Fig 3).

**Table 7:** Assessing the Quality of Studies.

Tele-SLT	Author, Year of publication	Study Design	Demographic variables	Aphasia variables	Telehealth characteristics	Data collection	Overall rating
**Assessment methods**	Choi, Y.H., et al., 2015 [30]	**High**	*Low*	**High**	**High**	*Low*	*Low*
	Guo, Y.E., et al., 2017 [44]	*Low*	**High**	**High**	**High**	Moderate	*Low*
	Altaib, M. K., et al., 2023 [31]	Moderate	**High**	**High**	**High**	Moderate	Moderate
**Synchronous** **training methods**	Agostini, M., et al., 2014 [32]	*Low*	**High**	Moderate	**High**	*Low*	*Low*
	Furnas, D. W., et al., 2014 [33]	*Low*	**High**	**High**	Moderate	*Low*	*Low*
	Marshall, J., et al., 2016 [34]	**High**	Moderate	Moderate	Moderate	Moderate	Moderate
	Macoir, J., et al., 2017 [35]	*Low*	*Low*	Moderate	**High**	*Low*	*Low*
	Pitt, R., et al., 2017 [36]	*Low*	Moderate	Moderate	**High**	**High**	*Low*
	Pitt, R., et al., 2019 [37]	*Low*	Moderate	**High**	**High**	**High**	*Low*
	Pitt, R., et al., 2019 [38]	*Low*	*Low*	*Low*	**High**	**High**	*Low*
	Grechuta, K., et al., 2019 [39]	Moderate	Moderate	**High**	**High**	Moderate	Moderate
	Giachero, A., et al., 2020 [40]	**High**	**High**	Moderate	**High**	**High**	**High**
	Grechuta, K., et al., 2020 [41]	*Low*	Moderate	**High**	**High**	Moderate	*Low*
	Øra, H. P., et al., 2020 [42]	**High**	**High**	Moderate	**High**	**High**	**High**
	Cruice, M., et al., 2021 [43]	*Low*	*Low*	Moderate	**High**	Moderate	*Low*
**Asynchronous training methods**	Ksts, R. C., et al., 1997 [45]	**High**	**High**	Moderate	*Low*	*Low*	*Low*
	Cherney, L. R., 2008 [46]	*Low*	Moderate	**High**	Moderate	Moderate	*Low*
	Manheim, L. M., 2009 [47]	*Low*	**High**	Moderate	Moderate	*Low*	*Low*
	Archibald, L. M. D., et al., 2009 [48]	*Low*	**High**	**High**	**High**	Moderate	*Low*
	Cherney, L.R., 2010 [49]	**High**	*Low*	**High**	Moderate	*Low*	*Low*
	Palmer, R., et al., 2012 [50]	**High**	*Low*	**High**	Moderate	Moderate	*Low*
	van Vuuren, S., et al., 2014 [51]	*Low*	*Low*	Moderate	Moderate	*Low*	*Low*
	Kurland, J., et al., 2014 [52]	*Low*	Moderate	**High**	**High**	*Low*	*Low*
	Cherney, L.R., et al., 2015 [53]	*Low*	**High**	**High**	Moderate	*Low*	*Low*
	Choi, Y. H., et al., 2016 [54]	*Low*	**High**	**High**	Moderate	*Low*	*Low*
	Kurland, J., et al., 2018 [55]	*Low*	**High**	**High**	Moderate	*Low*	*Low*
	Zhou, Q., et al., 2018 [56]	**High**	*Low*	**High**	Moderate	*Low*	*Low*
	Cherney, L.R., et al., 2019 [57]	*Low*	**High**	**High**	Moderate	*Low*	*Low*
	Maresca, G., et al., 2019 [58]	**High**	**High**	*Low*	**High**	*Low*	*Low*
	Palmer, R., et al., 2019 [59]	**High**	Moderate	**High**	Moderate	**High**	Moderate
	Spaccavento, S., et al., 2021 [60]	Moderate	Moderate	Moderate	**High**	Moderate	Moderate
	Cherney, L. R., et al., 2021 [61]	**High**	**High**	**High**	**High**	*Low*	*Low*
	Braley, M., et al., 2021 [62]	**High**	**High**	**High**	**High**	Moderate	**High**
	Cherney, L. R., et al., 2022 [63]	*Low*	**High**	**High**	Moderate	*Low*	*Low*
**Combined Training method**	Woolf, C., et al., 2015 [64]	Moderate	*Low*	*Low*	**High**	*Low*	*Low*

Note: Bold indicates high assessment, italic indicates low assessment, and underline indicates moderate assessment. This research quality assessment tool is based on Teti et al (2023) [21] and Salis et al. (2021) [26] draws upon the information from the McMaster University Health Evidence Quality Assessment Tool Dictionary. For a study to receive an overall high rating (excluding low ratings), it must score highly on at least four out of the five criteria.

### Training effects of Tele-SLT

**Fig 4:**
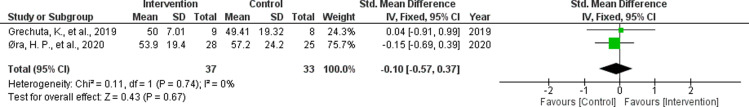
Results of a Meta-Analysis of the Effects of Synchronous Training on Communication Outcomes.

**Fig 5:**
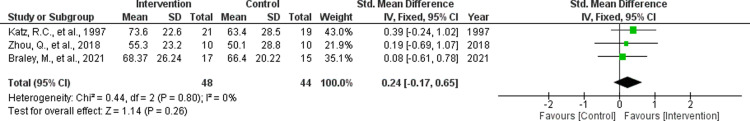
Results of a Meta-Analysis of the Effects of Asynchronous Training on the Western Aphasia Battery-Aphasia Quotient.

**Fig 6:**
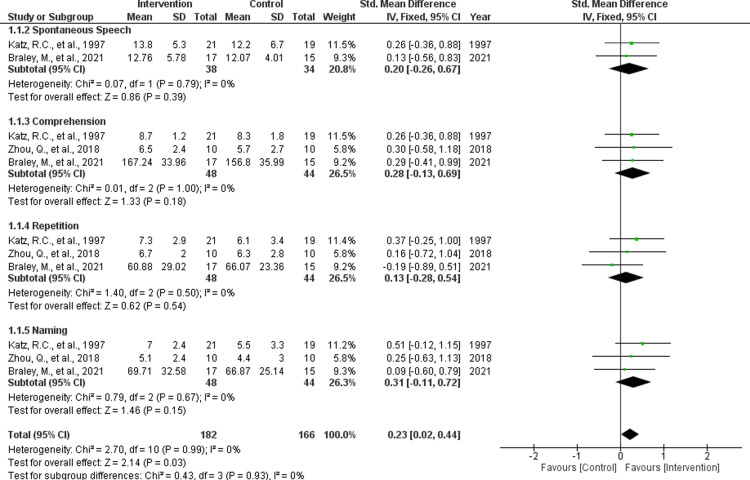
Results of a Meta-Analysis of the Effects of Asynchronous Training on the Western Aphasia Battery Subscales, Western Aphasia Battery Revised Subscales.

We performed a meta-analysis of Tele-SLT training effects using data from four RCTs. Two studies examined synchronous training using the CAL and CETI as outcome measures [39,42], and three examined asynchronous training using the WAB, WAB-R as the outcome measure [45,56,62]. For synchronous training, there was no significant difference between the intervention and control groups in combined CAL and CETI scores (fixed-effects model, SMD =  -0.10, 95% CI =  -0.57 to 0.37, *P* >  0.05) (Fig 4). For asynchronous training, analysis of the WAB-AQ showed no significant difference between groups (fixed-effects model, SMD =  0.24, 95% CI =  -0.17 to 0.65, *P* >  0.05) (Fig 5). However, a significant difference was found in the combined WAB subscales (Spontaneous speech, Comprehension, Repetition, Naming) (fixed-effects model, SMD =  0.23, 95% CI =  0.02 to 0.44, *P* <  0.05). Despite the significant result for the combined subscales, no significant differences were observed for individual subscales: Spontaneous speech (fixed-effects model, SMD =  0.20, 95% CI =  -0.26 to 0.67, *P* >  0.05), Comprehension (fixed-effects model, SMD =  0.28, 95% CI =  -0.13 to 0.69, *P* >  0.05), Repetition (fixed-effects model, SMD =  0.13, 95% CI =  -0.28 to 0.54, *P* >  0.05) and Naming (fixed-effects model, SMD =  0.31, 95% CI =  -0.11 to 0.72, *P* >  0.05) (Fig 6)

## Discussion

### Findings of this Tele-SLT review

This review investigated Tele-SLT support and delivery methods for PWA. Our findings reveal a growing body of research examining the use of specialized software for Tele-SLT, particularly for individuals with chronic PWA; many studies demonstrate its benefits. However, three key challenges emerged. First, research on tele-assessment is scarce compared to research on tele-training. Second, there is insufficient research on synchronous remote SLT compared to asynchronous remote SLT, and RCTs have yet to demonstrate a significant training effect for synchronous interventions compared to asynchronous interventions. Third, the overall methodological quality of Tele-SLT research is low. These findings suggest that the availability of diverse Tele-SLT approaches may be limited, and the efficacy of Tele-SLT, particularly synchronous training, warrants further investigation. As the demand for Tele-SLT increases, addressing these challenges is crucial.

### Bias in the number of studies on the content of support in Tele-SLT

Only three studies (8.57%) involving tele-assessments were included in this study, highlighting the challenges associated with conducting tele-assessments. Specifically, it may be difficult to remotely determine whether an individual is PWA and to obtain detailed information about them, potentially hindering the provision of tele-training [65]. A significant challenge is the influence of digital device functionality and the environment on the interpretation of PWA outcomes. For instance, in videoconferencing communication assessments, there is a concern that microphones may fail to capture unintelligible or faint utterances caused by speech or language disorders, making it challenging to derive clinical insights into spoken language [66]. Furthermore, loss of video data has been noted due to volume changes associated with the PWA’s sitting position and inadequate computer capacity, even when the microphone’s audio input is normal [66]. Therefore, implementing tele-assessments may necessitate selecting an assessment modality based on the severity of the PWA’s aphasia and the device’s capabilities, as well as verifying the assessment environment in advance [66]. From clinical or research perspectives, establishing such protocols and checklists is essential [67]. In the context of Tele-SLT, utilizing conversation recording protocols from Aphasiabank, an online platform for sharing PWA data, may be beneficial [68]. If these procedures are made accessible to SLP, other professionals, and PWA in a format compatible with Tele-SLT, it is likely that tele-evaluation will gain popularity as a user-friendly approach.

### Limited research on synchronous Tele-SLT delivery

Our review found that only 14 of the 35 included studies (40.00%) investigated synchronous Tele-SLT, suggesting potential challenges in its implementation. This scarcity may indicate a lack of strategies for addressing communication, QOL, and well-being in PWA through synchronous Tele-SLT [69–71]. Such support is often associated with, and facilitated by, the interactive communication inherent in synchronous interventions. Interactive communication promotes social participation in PWA [72], which is directly linked to QOL and well-being [5]. Therefore, synchronous Tele-SLT may be crucial for supporting social participation and QOL in PWA, and addressing barriers to its implementation is essential.

Potential barriers may include concerns about privacy and confidentiality, as well as a lack of ethical and technical support for using video conferencing in synchronous Tele-SLT [73]. Developing guidelines and checklists regarding privacy, confidentiality, and the use of communication technology for PWA could help mitigate these issues [66]. Without standardized practices for video conferencing in SLT, the expansion of synchronous Tele-SLT may be hindered, and the inconclusive training effects suggested by our meta-analysis may remain unresolved. Without addressing these challenges, we risk missing opportunities to provide effective SLT to PWA who have faced increased difficulties with social participation and daily living since the onset of the COVID-19 pandemic.

### Quality of Tele-SLT studies

The quality of Tele-SLT studies has been consistently rated as low. The main reasons for this can be summarized as follows: poor baseline data due to the lack of standardized assessment methods for PWA, as noted in the ‘Data Collection’ section, and an undefined target group and insufficient sample size, as mentioned in the ‘Study Design’ section. Regarding the former, the shortcomings of standardized assessment methods in SLT have been documented [74], and adapting traditional tests for Tele-SLT, as has been done with the WAB [75], may be necessary. With regard to the latter, barriers such as inattention and poor auditory comprehension in PWA have hindered their participation in telemedicine [66]. This is why maintaining and increasing sample sizes in studies involving PWA has been challenging. Although some suggest that patient sampling is easier in telerehabilitation [76], this may not hold true for PWA. Therefore, study designs must consider the cognitive and language functions of PWA to ensure an adequate sample size.

### Future directions in the post-COVID-19 era

The recent COVID-19 pandemic has increased the challenges of delivering in-person SLT, which has led to a surge in global interest in Tele-SLT. Establishing effective tele-assessment and synchronous training methods, which our review identified as areas in need of further development, is a high priority. Widespread implementation of Tele-SLT has the potential to expand access to SLT for a greater number of PWA. To achieve this, it is crucial to determine which types of Tele-SLT (assessment, training, synchronous, asynchronous, or combined) are most effective for specific PWA profiles. Therefore, improving the quantity and quality of research across all areas of Tele-SLT, including the development of tele-assessment tools and synchronous training protocols specifically designed for PWA, is essential.

One approach to address this challenge is to promote the development and use of specialized software for PWA. Such software was utilized in 68.57% (n =  24/35) of the studies included in this review. Specialized software can facilitate the management of patient information in rehabilitation settings and, under appropriate conditions, enable high-quality audio and video transmission [19]. Furthermore, integrating software into Tele-SLT practice is thought to reduce the burden on speech-language pathologists and promote wider adoption of Tele-SLT [77]. Therefore, developing and disseminating PWA-specific software could be instrumental in expanding access to and strengthening the evidence base for Tele-SLT.

### Limitations of this review

This review has five main limitations. First, the failure to use specific checklists for the quantitative quality assessment of the included studies may introduce uncertainty regarding the consistency of the study quality in this scoping review design. Second, while a comprehensive literature search was conducted, the exclusion of articles in languages other than English and Japanese, as well as gray literature and manual searches of specific databases, may have led to selection bias. Third, the exclusion of articles in languages other than English and Japanese in the meta-analysis limited the number of studies included for analysis, and we could not conduct subgroup analyses based on participant characteristics or details of interventions. Therefore, caution is needed when interpreting the results of the meta-analysis. Fourth, as we focused on studies that included multiple participants with aphasia, single-case study designs were excluded. Finally, as with all database searches, it is impossible to completely eliminate publication bias [78].

## Conclusion

In this study, we reviewed assessment and training methods in Tele-SLT for PWA and categorized them into synchronous, asynchronous, and combined methods. Despite an initial search of 1,484 articles from five databases, only 35 met our strict inclusion criteria. This finding underscores the scarcity of research on tele-assessment and synchronous training and highlights the overall poor quality of available studies. Given the increasing societal involvement of PWA, research in these areas is particularly critical in the post-COVID-19 era, and continued research is necessary. However, all studies showed valid results, and Tele-SLT methodologies, including digital programs dedicated to PWA, appear to be indicators of future solutions to the problem.

## Supporting information

S1 Table Search formula used in this study.
(DOCX)

S2 Table Criteria for evaluating the quality of studies.
(DOCX)

S3 Table Results by evaluator and final evaluation of the secondary screening inclusion articles.
(DOCX)
