# Supplementary material for: Current status of Tele-speech language therapy by type and support for patients with post-stroke aphasia: A scoping review
Source: PLoS One. 2025 Mar 25;20(3):e0319805. doi: 10.1371/journal.pone.0319805 (PMC11936174; doi:10.1371/journal.pone.0319805)
Supplement: S1 Table — (DOCX) [file pone.0319805.s001.docx]

Table S1 Search formula used in this study

| **Components** | **Search items** | **Results** |
| --- | --- | --- |
| **PubMed** | | |
| #1 | | |
| #2 | "Aphasia"[MH] OR "aphasi*"[TW] OR "anomi*"[TW] OR "alogi*"[TW] OR "anepi*"[TW] OR "dysphasi*"[TW] OR "word deafness"[TW] OR lichtheim[TW] | 23,599 |
| #3 | "Telemedicine"[MH] OR "telerehabilit*"[TW] OR "telepractice"[TIAB] OR "teleconf*"[TIAB] OR (("rehabilit*"[TIAB] OR "therap*"[TIAB] OR "training"[TIAB]) AND ("tele*"[TIAB] OR "online"[TIAB] OR "remote"[TIAB] OR "video"[TIAB] OR "virtual*"[TIAB] OR "web"[TIAB] OR "computer"[TIAB] OR "screen to screen"[TIAB])) | 219,455 |
| #4 | #1 AND #2 | 455 |
| Embase | | |
| s1 | | |
| s2 | EMB.EXACT("aphasia") OR ab(aphasi*) OR ab(anomi*) OR ab(alogi*) OR ab(anepi*) OR ab(dysphasi*) OR ab("word deafness") OR ab(logagnosi*) OR ab(logamnesi*) OR ab(logastheni*) | 41,317 |
| s3 | EMB.EXACT("telerehabilitation") OR EMB.EXACT("telemedicine") | 46,342 |
| s4 | ab(telerehabilitation OR telemedicine OR telepractice OR (rehabilit* AND (tele* OR online OR remote OR video OR virtual OR Web OR computer))) OR ti(telerehabilitation OR telemedicine OR telepractice OR (rehabilit* AND (tele* OR online OR remote OR video OR virtual OR Web OR computer))) | 48,633 |
| s5 | S3 OR S2 | 72,600 |
| s6 | S4 AND S1 | 513 |
| PsycINFO | | |
| S1 | SU.EXACT.EXPLODE("Aphasia") OR ab(aphasi* OR anomi* OR alogi* OR anepi* OR dysphasi* OR "word deafness" OR lichtheim) OR ti(aphasi* OR anomi* OR alogi* OR anepi* OR dysphasi* OR "word deafness" OR lichtheim) | 25,950 |
| S2 | SU.EXACT.EXPLODE("Telemedicine") OR ab(telerehabilitation OR telemedicine OR telepractice OR (rehabilit* AND (tele* OR online OR remote OR video OR virtual OR Web OR computer))) OR ti(telerehabilitation OR telemedicine OR telepractice OR (rehabilit* AND (tele* OR online OR remote OR video OR virtual OR Web OR computer))) | 18,666 |
| S3 | S2 AND S1 | 202 |
| Cochrane | | |
| #1 | MeSH descriptor: [Aphasia] explode all trees | 596 |
| #2 | (aphasia):ti,ab,kw OR (alogia):ti,ab,kw OR (anepia):ti,ab,kw OR (dysphasia):ti,ab,kw OR (lichtheim):ti,ab,kw | 2,136 |
| #3 | #1 OR #2 | 2,136 |
| #4 | MeSH descriptor: [Telemedicine] explode all trees | 4,006 |
| #5 | (telerehabilitation):ti,ab,kw OR (telemedicine):ti,ab,kw OR (telepractice):ti,ab,kw OR (teleconf*):ti,ab,kw OR ((rehabilit* OR therap* OR training) AND (tele* OR online OR remote OR video OR virtual* OR web OR computer OR "screen-to-screen")):ti,ab,kw | 72,253 |
| #6 | #4 OR #5 | 72,362 |
| #7 | #3 AND #6 | 286 |

| Ichushi | | |
| --- | --- | --- |
| #1 | 失語症/TH or 失語/TA or 失名辞/TA or aphasia/TA or anomia/TA or alogia/TA or anepia/TA or dysphasia/TA or logagnosia/TA or logamnesia/TA or logasthenia/TA | 16,467 |
| #2 | @"遠隔医療"/TH or 遠隔リハビリテーション/TH or telerehabilitation/TA or "tele-rehabilitation"/TA or "tele rehabilitation"/TA or ((リハビリ/TA or 訓練/TA or トレーニング/TA or 療法/TA or rehabilit*/TA or training/TA or therap*/TA) and (遠隔/TA or リモート/TA or オンライン/TA or バーチャル/TA or ヴァーチャ | 13,862 |
| #3 | #1 and #2 | 28 |
