# Supplementary material for: Current status of Tele-speech language therapy by type and support for patients with post-stroke aphasia: A scoping review
Source: PLoS One. 2025 Mar 25;20(3):e0319805. doi: 10.1371/journal.pone.0319805 (PMC11936174; doi:10.1371/journal.pone.0319805)
Supplement: S2 Table — (DOCX) [file pone.0319805.s002.docx]

Table S2. Criteria for evaluating the quality of studies

| Appraisal Categories | High | Moderate | Low |
| --- | --- | --- | --- |
| Study Design | Training: RCT or quasi-RCT with ≥ 10 participants in each group.  Evaluation: Cross-sectional study with ≥ 20 participants in each group. | Training: RCT or quasi-RCT with < 10 participants in each group.  Evaluation: Cross-sectional study with < 20 participants in each group. | Unable to obtain a moderate standard. |
| Demographic variables | At least five demographic variables (e.g., age, gender, education, handedness, language, socioeconomic status, relevant comorbidities) should be reported. | At least four demographic variables must be reported. |  |
| Aphasia variables | Language proficiency (e.g., fluency), severity of aphasia, and time since onset of aphasia must all be reported. | Report: Two Variables of Aphasia |  |
| Tele-SLT characteristics | Report the platform used (e.g., software), the device utilized (e.g., computer, tablet), internet access method (e.g., Wi-Fi), and three modes of audio and visual transmission/reception (e.g., headphones, speakers) | Report: Two Tele-SLT characteristics |  |
| Data collection | The table incorporates standardized outcome measures, at least four total metrics, including performance-based language measures, patient/caregiver perceptions (e.g., communication quality, telehealth feasibility), and clinician feedback on telehealth feasibility. | The table includes one standardized measure and at least three others, representing performance-based language, patient/caregiver feedback, or clinician telehealth feedback. |  |

Note: This assessment tool is adapted from Teti et al. (2023) [21] and Salis et al. (2021) [26] based on the McMaster University dictionary. A study needs high scores in 4 out of 5 criteria for a high rating, with no low scores allowed for a medium rating. “Standardized” refers to measures that are commonly used in clinical or research settings, have documented normative data, and have an established evidence base
