# Supplementary material for: Current status of Tele-speech language therapy by type and support for patients with post-stroke aphasia: A scoping review
Source: PLoS One. 2025 Mar 25;20(3):e0319805. doi: 10.1371/journal.pone.0319805 (PMC11936174; doi:10.1371/journal.pone.0319805)
Supplement: S3 Table — (DOCX) [file pone.0319805.s003.docx]

Table S3. Results by evaluator and final evaluation of the secondary screening inclusion articles

| No. | Secondary screening inclusion articles | Evaluator 1 (YK) | Evaluator 2 (AY) | Final decision | Reasons for the decision to exclude |
| --- | --- | --- | --- | --- | --- |
| 1 | Trebilcock M, et al. Pilot trial of the online implementation intervention Aphasia Nexus: Connecting Evidence to Practice. Int J Speech Lang Pathol. 2024; 26: 16-27. (Published online: 23 Dec 2022) | Exclude | Exclude | Exclude | Different study designs |
| 2 | de Grosbois J, et al. Asynchronous, online spaced-repetition training alleviates word-finding difficulties in aphasia. Neuropsychol Rehabil. 2023; 10: 1672-1692. (Published online: 15 Nov 2022) | Exclude | Exclude | Exclude | Different target group |
| 3 | Quique YM, et al. Applying adaptive distributed practice to self-managed computer-based anomia treatment: A single-case experimental design. J Commun Disord. 2022; 106249. | Exclude | Exclude | Exclude | Different methods of support and intervention |
| 4 | Liu M, et al. Improvement in language function in patients with aphasia using computer-assisted executive function training: A controlled clinical trial. PM R. 2022; 14: 913-921. | Exclude | Exclude | Exclude | Different methods of support and intervention |
| 5 | Trebilcock M, et al. Development of an online implementation intervention for aphasia clinicians to increase the intensity and comprehensiveness of their service. Disabil Rehabil. 2022; 44: 4629-4638. | Exclude | Exclude | Exclude | Different study designs |
| 6 | Rohde A, et al. Inter-rater reliability, intra-rater reliability and internal consistency of the Brisbane Evidence-Based Language Test. Disabil Rehabil. 2022; 44: 637-645. | Exclude | Exclude | Exclude | Different target group |
| 7 | Spaccavento S, et al. Effects of computer-based therapy versus therapist-mediated therapy in stroke-related aphasia: Pilot non-inferiority study. J Commun Disord. 2021; 94: 106158. | Exclude | Include | Include | - |
| 8 | Jacobs M, et al. Estimating the cost and value of functional changes in communication ability following telepractice treatment for aphasia. PLoS One. 2021; 16: e0257462. | Exclude | Exclude | Exclude | Different target group |
| 9 | Cherney LR, et al. Web-based Oral Reading for Language in Aphasia (Web ORLA(®)): A pilot randomized control trial. Clin Rehabil. 2021; 35: 976-987. | Include | Include | Include | - |
| 10 | Harrison M, et al. Factors Associated With Adherence to Self-Managed Aphasia Therapy Practice on a Computer-A Mixed Methods Study Alongside a Randomized Controlled Trial. Front Neurol. 2020; 11: 582328. | Exclude | Exclude | Exclude | Different study designs |
| 11 | Giachero A, et al. Conversational Therapy through Semi-Immersive Virtual Reality Environments for Language Recovery and Psychological Well-Being in Post Stroke Aphasia. Behav Neurol. 2020: 2846046. | Include | Include | Include | - |
| 12 | Dekhtyar M, et al. Videoconference Administration of the Western Aphasia Battery–Revised: Feasibility and Validity. Am J Speech Lang Pathol. 2020; 29: 673-687. | Exclude | Exclude | Exclude | Different target group |
| 13 | Cherney LR, et al. Optimising recovery in aphasia: Learning following exposure to a single dose of computer-based script training. Int J Speech Lang Pathol. 2019; 21: 448-458. | Include | Include | Include | - |
| 14 | Maresca G, et al. Toward Improving Poststroke Aphasia: A Pilot Study on the Growing Use of Telerehabilitation for the Continuity of Care. J Stroke Cerebrovasc Dis. 2019; 28: 104303. | Include | Include | Include | - |
| 15 | Kerry SJ, et al. How Does iReadMore Therapy Change the Reading Network of Patients with Central Alexia? J Neurosci. 2019; 39: 5719-5727. | Exclude | Exclude | Exclude | Different target group |
| 16 | Marshall J, et al. Technology-enhanced writing therapy for people with aphasia: results of a quasi-randomized waitlist controlled study. Int J Lang Commun Disord. 2019; 54: 203-220. | Exclude | Exclude | Exclude | Different methods of support and intervention |
| 17 | Klischies D, et al. Evaluation of Deep Clustering for Diarization of Aphasic Speech. Stud Health Technol Inform. 2019; 260: 81-88. | Exclude | Exclude | Exclude | Different publication classifications |
| 18 | Wall KJ, et al. Using technology to overcome the language barrier: the Cognitive Assessment for Aphasia App. Disabil Rehabil. 2018; 40: 1333-1344. | Exclude | Exclude | Exclude | Different study designs |
| 19 | Kurland J, et al. Effects of a Tablet-Based Home Practice Program With Telepractice on Treatment Outcomes in Chronic Aphasia. J Speech Lang Hear Res. 2018; 61: 1140-1156. | Include | Include | Include | - |
| 20 | Kesav P, et al. Effectiveness of speech language therapy either alone or with add-on computer-based language therapy software (Malayalam version) for early post stroke aphasia: A feasibility study. J Neurol Sci. 2017; 380: 137-141. | Exclude | Exclude | Exclude | Different methods of support and intervention |
| 21 | Macoir J, et al. In-Home Synchronous Telespeech Therapy to Improve Functional Communication in Chronic Poststroke Aphasia: Results from a Quasi-Experimental Study. Telemed J E Health. 2017; 23: 630-639. | Include | Include | Include | - |
| 22 | Roper A, et al. Benefits and Limitations of Computer Gesture Therapy for the Rehabilitation of Severe Aphasia. Front Hum Neurosci. 2016; 10: 595. | Exclude | Exclude | Exclude | Different methods of support and intervention |
| 23 | Marshall J, et a. Evaluating the Benefits of Aphasia Intervention Delivered in Virtual Reality: Results of a Quasi-Randomised Study. PLoS One. 2016; 11: e0160381. | Include | Include | Include | - |
| 24 | Ruiter MB, et al. An Exploratory Investigation of E-Rest: Teletherapy for Chronically Aphasic Speakers. Int J Telerehabil. 2016; 8: 21-28. | Exclude | Exclude | Exclude | Different study designs |
| 25 | Woolf C, et al. A comparison of remote therapy, face to face therapy and an attention control intervention for people with aphasia: a quasi-randomised controlled feasibility study. Clin Rehabil. 2016; 30: 359-373. | Exclude | Exclude | Exclude | Different methods of support and intervention |
| 26 | Varley R, et al. Self-Administered Computer Therapy for Apraxia of Speech: Two-Period Randomized Control Trial With Crossover. Stroke. 2016; 47: 822-828. | Include | Exclude | Exclude | Different target group |
| 27 | van Vuuren S, et al. A Virtual Therapist for Speech and Language Therapy. Intell Virtual Agents. 2014; 8637:438-448. | Include | Include | Include | - |
| 28 | Latimer NR, et al. Cost-utility of self-managed computer therapy for people with aphasia. Int J Technol Assess Health Care. 2013; 29 :402-409. | Exclude | Exclude | Exclude | Different study designs |
| 29 | Hussmann K, et al. Computer-assisted analysis of spontaneous speech: quantification of basic parameters in aphasic and unimpaired language. Clin Linguist Phon. 2012; 26: 661-680. | Exclude | Exclude | Exclude | Different target group |
| 30 | Cherney LR, et al. Computer-based script training for aphasia: emerging themes from post-treatment interviews. J Commun Disord. 2011; 44: 493-501. | Exclude | Exclude | Exclude | Different study designs |
| 31 | Cherney LR. Oral reading for language in aphasia (ORLA): evaluating the efficacy of computer-delivered therapy in chronic nonfluent aphasia. Top Stroke Rehabil. 2010; 17: 423-431. | Include | Include | Include | - |
| 32 | Manheim LM, et al. Patient-reported changes in communication after computer-based script training for aphasia. Arch Phys Med Rehabil. 2009; 90: 623-627. | Include | Include | Include | - |
| 33 | Palsbo SE. Equivalence of functional communication assessment in speech pathology using videoconferencing. J Telemed Telecare. 2007; 13: 40-43. | Exclude | Include | Exclude | Different target group |
| 34 | Laganaro M., et al. Acquired alexia in multilingual aphasia and computer-assisted treatment in both languages: issues of generalisation and transfer. Folia Phoniatr Logop. 2002; 53: 135-144. | Exclude | Exclude | Exclude | Different study designs |
| 35 | Aftonomos LB, et al. Improving outcomes for persons with aphasia in advanced community-based treatment programs. Stroke. 1999; 30: 1370-1379. | Exclude | Exclude | Exclude | Different target group |
| 36 | Katz RC, et al. The efficacy of computer-provided reading treatment for chronic aphasic adults. J Speech Lang Hear Res. 1997; 40: 493-507. | Include | Include | Include | - |
| 37 | De Luca R, et al. Virtual reality as a new tool for the rehabilitation of post-stroke patients with chronic aphasia: an exploratory study. Aphasiology. 2023; 37: 249-259. | Exclude | Exclude | Exclude | Different methods of support and intervention |
| 38 | Macedonia M, et al. Gesture based word (re)acquisition with a virtual agent in augmented reality: A preliminary study. Annu Rev CyberTher Telemed. 2022; 20: 83-86. | Exclude | Exclude | Exclude | Different target group |
| 39 | Cruice M, et al. Preliminary outcomes from a pilot study of personalised online supported conversation for participation intervention for people with Aphasia. Aphasiology. 2021; 35: 1293-1317. | Include | Include | Include | - |
| 40 | Grasso SM, et al. A tablet-based home practice program paired with telepractice promotes maintenance and learning of objects and actions in individuals with chronic aphasia. Evid Base Commun Assess Interv. 2019; 13: 171-176. | Exclude | Exclude | Exclude | Different target group |
| 41 | Amaya A, et al. Receiving aphasia intervention in a virtual environment: the participants’ perspective. Aphasiology. 2018; 32: 538-558. | Include | Include | Include | - |
| 42 | Hess DC, et al. Stroke telepresence: Removing all geographic barriers. Neurology. 2011; 76: 1121-1123. | Exclude | Exclude | Exclude | Different publication classifications |
| 43 | Steele RD, et al. Outcome improvements in persons with chronic global aphasia following the use of a speech-generating device. Acta Neuropsychologica. 2010; 8: 342-359. | Exclude | Exclude | Exclude | Different target group |
| 44 | Hill MAJ, et al. The effects of aphasia severity on the ability to assess language disorders via telerehabilitation. Aphasiology. 2009; 23: 627-642. | Exclude | Exclude | Exclude | Different target group |
| 45 | Bruckert R, et al. The use of a computer driven videodisc for the assessment and rehabilitation of aphasia. Aphasiology. 1989; 3: 473-478. | Exclude | Exclude | Exclude | Different publication classifications |
| 46 | De Luca R, et al. Computerized Training in Poststroke Aphasia: what About the Long-Term Effects? A Randomized Clinical Trial. J Stroke Cerebrovasc Dis. 2018; 27: 2271-2276. | Exclude | Exclude | Exclude | Different target group |
| 47 | Automatic Assessment of Aphasic Speech Sensed by Audio Sensors for Classification into Aphasia Severity Levels to Recommend Speech Therapies. | Exclude | Exclude | Exclude | Different target group |
| 48 | Guo YE, et al. Assessment of Aphasia Across the International Classification of Functioning, Disability and Health Using an iPad-Based Application. Telemed J E Health. 2017; 23: 313-326. | Include | Include | Include | - |
| 49 | Choi YH, et al. A Telescreening Tool to Detect Aphasia in Patients with Stroke. Telemed J E Health. 2015; 21: 729-734. | Include | Exclude | Include | - |
| 50 | Cherney LR, et al. Complexity and Feedback During Script Training in Aphasia: A Feasibility Study. Arch Phys Med Rehabil. 2022;103: 205-214. | Include | Include | Include | - |
| 51 | Musso M, et al. Aphasia Recovery by Language Training Using a Brain–Computer Interface: A Proof-of-Concept Study. Brain Commun. 2022; 4: fcac008. | Exclude | Exclude | Exclude | Different methods of support and intervention |
| 52 | Velez JA, et al. A Visual Interactive Narrative Intervention (VINI) for Aphasia Education: Can Digital Applications Administer Augmented Input to Educate Stroke Survivors with Aphasia? Patient Educ Couns. 2021; 104: 2536-2543. | Exclude | Exclude | Exclude | Different methods of support and intervention |
| 53 | Braley M, et al. A Virtual, Randomized, Control Trial of a Digital Therapeutic for Speech, Language, and Cognitive Intervention in Post-stroke Persons With Aphasia. Front Neurol. 2021;12:626780. | Include | Include | Include | - |
| 54 | Gallée J, et al. The Application of Lexical Retrieval Training in Tablet-Based Speech-Language Intervention. Front Neurol. 2020;11:583246. | Exclude | Exclude | Exclude | Different target group |
| 55 | Grechuta K, et al. Multisensory Cueing Facilitates Naming in Aphasia. J NeuroEng Rehabil. 2020;17:122. | Include | Include | Include | - |
| 56 | Øra HP, et al. The Effect of Augmented Speech-Language Therapy Delivered by Telerehabilitation on Poststroke Aphasia—A Pilot Randomized Controlled Trial. Clin Rehabil. 2020; 34: 918-930​. | Include | Include | Include | - |
| 57 | Pitt R, et al. The Impact of the Telerehabilitation Group Aphasia Intervention and Networking Programme on Communication, Participation, and Quality of Life in People with Aphasia. Int J Speech Lang Pathol. 2018; 20: 1-11​. | Include | Include | Include | - |
| 58 | Palmer R, et al. Self-managed, Computerised Speech and Language Therapy for Patients with Chronic Aphasia Post-Stroke Compared with Usual Care or Attention Control (Big CACTUS): A Multicentre, Single-Blinded, Randomised Controlled Trial. Lancet Neurol. 2019; 18: 821-833​. | Include | Include | Include | - |
| 59 | Grechuta K, et al. Augmented Dyadic Therapy Boosts Recovery of Language Function in Patients With Nonfluent Aphasia: A Randomized Controlled Trial. Stroke. 2019; 50: 1270-1274. | Include | Include | Include | - |
| 60 | Pitt R, et al. The development and feasibility of an online aphasia group intervention and networking program – TeleGAIN. Int J Speech Lang Pathol. 2019; 21: 23-36. | Include | Include | Include | - |
| 61 | Rhodes NC, et al. Script Training Using Telepractice With Two Adults With Chronic Non-Fluent Aphasia. Int J Telerehabil. 2018; 10: 89-104. | Exclude | Exclude | Exclude | Different target group |
| 62 | Zhou Q, et al. Telerehabilitation Combined Speech-Language and Cognitive Training Effectively Promoted Recovery in Aphasia Patients. Front Psychol. 2018; 9: 2312. | Include | Include | Include | - |
| 63 | Pitt R, et al. The Feasibility of Delivering Constraint-Induced Language Therapy via the Internet. Digit Health. 2017; 3: 2055207617718767 | Include | Include | Include | - |
| 64 | Thiel L, et al. Promoting Linguistic Complexity, Greater Message Length, and Ease of Engagement in Email Writing in People with Aphasia: Initial Evidence from a Study Utilizing Assistive Writing Software. Int J Lang Commun Disord. 2016; 00: 1-19. | Exclude | Exclude | Exclude | Different methods of support and intervention |
| 65 | Hill AJ, et al. Refining an Asynchronous Telerehabilitation Platform for Speech-Language Pathology: Engaging End-Users in the Process. Front Hum Neurosci. 2016; 10: 640. | Exclude | Exclude | Exclude | Different study designs |
| 66 | Best W, et al. Conversation Therapy with People with Aphasia and Conversation Partners Using Video Feedback: A Group and Case Series Investigation of Changes in Interaction. Front Hum Neurosci. 2016;10:562. | Exclude | Exclude | Exclude | Different methods of support and intervention |
| 67 | Simic T, et al. A Usability Study of Internet-Based Therapy for Naming Deficits in Aphasia. Am J Speech Lang Pathol. 2016; 25: 581-593. | Exclude | Exclude | Exclude | Different study designs |
| 68 | Choi YH, et al. A Telerehabilitation Approach for Chronic Aphasia Following Stroke. Telemed J E Health. 2016; 22: 434-440​. | Include | Include | Include | - |
| 69 | Cherney LR, et al. Impact of Personal Relevance on Acquisition and Generalization of Script Training for Aphasia: A Preliminary Analysis. Am J Speech Lang Pathol. 2015;24: 913-922. | Include | Include | Include | - |
| 70 | Steele RD, et al. Combining Teletherapy and On-Line Language Exercises in the Treatment of Chronic Aphasia: An Outcome Study. Int J Telerehabil. 2014;6(2):3-1. | Exclude | Exclude | Exclude | Different methods of support and intervention |
| 71 | Kurland J, et al. iPractice: Piloting the Effectiveness of a Tablet-Based Home Practice Program in Aphasia Treatment. Semin Speech Lang. 2014; 35: 51-64. | Include | Include | Include | - |
| 72 | Agostini M, et al. Telerehabilitation in Poststroke Anomia. BioMed Res Int. 2014; 2014:706909. | Include | Include | Include | - |
| 73 | Goldberg S, et al. Script Training and Generalization for People With Aphasia. Am J Speech Lang Pathol. 2012; 21: 222-238. | Exclude | Exclude | Exclude | Different target group |
| 74 | Palmer R, et al. Computer Therapy Compared With Usual Care for People With Long-Standing Aphasia Poststroke: A Pilot Randomized Controlled Trial. Stroke. 2012; 43: 1904-1911. | Include | Include | Include | - |
| 75 | Dechêne L, et al. Simulated In-Home Teletreatment for Anomia: A Pilot Study. Int J Telerehabil. 2011; 3: 3-10. | Exclude | Exclude | Exclude | Different methods of support and intervention |
| 76 | Adrián JA, et al. Extending the Use of Spanish Computer-Assisted Anomia Rehabilitation Program (CARP-2) in People with Aphasia. J Commun Disord. 2011; 44: 666-677​. | Exclude | Exclude | Exclude | Different target group |
| 77 | Nicholas ML, et al. C-Speak Aphasia Alternative Communication Program for People with Severe Aphasia: Importance of Executive Functioning and Semantic Knowledge. Neuropsychol Rehabil. 2011; 21: 322-366. | Exclude | Exclude | Exclude | Different methods of support and intervention |
| 78 | Thompson CK, et al. Sentactics®: Computer-Automated Treatment of Underlying Forms in Agrammatic Aphasia. Aphasiology. 2010; 24: 1242-1266. | Exclude | Exclude | Exclude | Different target group |
| 79 | Archibald LM, et al. Implementation of Computer-Based Language Therapy in Aphasia. Ther Adv Neurol Disord. 2009; 2: 299-311. | Include | Include | Include | - |
| 80 | Cherney LR, et al. Novel Technology for Treating Individuals with Aphasia and Concomitant Cognitive Deficits. Top Stroke Rehabil. 2008; 15: 542–554. | Include | Include | Include | - |
| 81 | Laganaro M, et al. Computerised Treatment of Anomia in Acute Aphasia: Treatment Intensity and Training Size. Neuropsychol Rehabil. 2006; 16: 630-640. | Exclude | Exclude | Exclude | Different target group |
| 82 | Van de Sandt-Koenderman M, et al. A Computerised Communication Aid for People with Aphasia. Disabil Rehabil. 2005; 27: 529–533​. | Exclude | Exclude | Exclude | Different target group |
| 83 | Altaib MK, et al. A Feasibility Study of Teleassessment for People with Aphasia in Saudi Arabia and Kuwait. Aphasiology. 2023; 37: 1198–1222​. | Include | Include | Include | - |
| 84 | Zannino GD, et al. Picture and Spoken Word Presentation in Repetition Training for Anomia: Does Stimulus Order Matter? Evidence from a Computer-Based Telemedicine Approach. Aphasiology. 2020; 34: 275-299. | Exclude | Exclude | Exclude | Different target group |
| 85 | Furnas DW, et al. The Effect of Computerised Verb Network Strengthening Treatment on Lexical Retrieval in Aphasia. Aphasiology. 2014; 28: 401–420. | Include | Include | Include | - |
| 86 | Pedersen PM, et al. Improvement of Oral Naming by Unsupervised Computerised Rehabilitation. Aphasiology. 2001; 15: 151-169​. | Exclude | Exclude | Exclude | Different study designs |
| 87 | Duffy JR, et al. Telemedicine and the Diagnosis of Speech and Language Disorders. Mayo Clin Proc. 1997; 72: 1116-1122. | Exclude | Exclude | Exclude | Different target group |
| 88 | Bartlett MR, et al. Informativeness Ratings of Messages Created on an AAC Processing Prosthesis. Aphasiology. 2007; 21: 475-498.​ | Exclude | Exclude | Exclude | Different methods of support and intervention |
| 89 | Wade J, et al. Talk About IT: Views of People With Aphasia and Their Partners on Receiving Remotely Monitored Computer-Based Word Finding Therapy. Aphasiology. 2003; 17: 1031-1056​. | Exclude | Exclude | Exclude | Different study designs |
| 90 | Deloche G, et al. Confrontation Naming Rehabilitation in Aphasics: A Computerised Written Technique. Neuropsychol Rehabil. 1992; 2: 117-124.​ | Exclude | Exclude | Exclude | Different methods of support and intervention |
