# Supplementary material for: Current status of Tele-speech language therapy by type and support for patients with post-stroke aphasia: A scoping review
Source: PLoS One. 2025 Mar 25;20(3):e0319805. doi: 10.1371/journal.pone.0319805 (PMC11936174; doi:10.1371/journal.pone.0319805)
Supplement: Attachment — Submitted filename: Response letter.docx [file pone.0319805.s007.docx]

Response to the reviewer

Comment 1:

The manuscript has significantly improved, and the authors have addressed all the comments provided. The number of articles has increased, which offers more insightful information. Additionally, a meta-analysis has been incorporated into the study. However, the scope remains limited, as the focus was primarily on two languages and the inclusion criteria were quite strict. There is also potential for an increase in the number of studies included in the meta-analysis.

Response to the reviewer: Thank you for your valuable feedback on the revised manuscript, which includes additional studies and new analyses. We appreciate your insightful comments regarding the meta-analysis. Our initial stringent inclusion criteria resulted in a small sample size. Therefore, following your suggestion, we incorporated the Western Aphasia Battery-Revised (WAB-R) as an outcome measure and included the results from Braley M., et al. [62] in the analysis of asynchronous speech-language therapy (SLT).

While the addition of the WAB-R and the study by Braley et al. did not reveal a significant effect of asynchronous SLT on the Aphasia Quotient or the subtests of either the Western Aphasia Battery or the WAB-R, we did observe an overall effect. This finding remains consistent with our previous conclusion and is reflected in the Results section and Figures 5 and 6.

We acknowledge the limitations of the meta-analysis due to the small number of included studies and the potential lack of optimal standardization regarding inclusion criteria, outcome measures, and interventions provided to the control groups. We have added a statement addressing these limitations to the "Study Limitations" subsection of the Discussion.

**(Page 31–32, Line 267–284)**

**Training effects of Tele-SLT**

We performed a meta-analysis of Tele-SLT training effects using data from four RCTs. Two studies examined synchronous training using the CAL and CETI as outcome measures [39, 42], and three examined asynchronous training using the WAB, WAB-R as the outcome measure [45, 56, 62]. For synchronous training, there was no significant difference between the intervention and control groups in combined CAL and CETI scores (fixed-effects model, SMD = -0.10, 95% CI = -0.57 to 0.37, P > 0.05) (Figure 4). For asynchronous training, analysis of the WAB-AQ showed no significant difference between groups (fixed-effects model, SMD = 0.24, 95% CI = -0.17 to 0.65, P > 0.05) (Figure 5). However, a significant difference was found in the combined WAB subscales (Spontaneous speech, Comprehension, Repetition, Naming) (fixed-effects model, SMD = 0.23, 95% CI = 0.02 to 0.44, P < 0.05). Despite the significant result for the combined subscales, no significant differences were observed for individual subscales: Spontaneous speech (fixed-effects model, SMD = 0.20, 95% CI = -0.26 to 0.67, P > 0.05), Comprehension (fixed-effects model, SMD = 0.28, 95% CI = -0.13 to 0.69, P > 0.05), Repetition (fixed-effects model, SMD = 0.13, 95% CI = -0.28 to 0.54, P > 0.05) and Naming (fixed-effects model, SMD = 0.31, 95% CI = -0.11 to 0.72, P > 0.05) (Fig 6)

**(Page 37–38, Line 385–398)**

**Limitations of this Review**

This review has five main limitations. First, the failure to use specific checklists for the quantitative quality assessment of the included studies may introduce uncertainty regarding the consistency of the study quality in this scoping review design. Second, while a comprehensive literature search was conducted, the exclusion of articles in languages other than English and Japanese, as well as gray literature and manual searches of specific databases, may have led to selection bias. Third, the exclusion of articles in languages other than English and Japanese in the meta-analysis limited the number of studies included for analysis, and we could not conduct subgroup analyses based on participant characteristics or details of interventions. Therefore, caution is needed when interpreting the results of the meta-analysis. Fourth, as we focused on studies that included multiple participants with aphasia, single-case study designs were excluded. Finally, as with all database searches, it is impossible to completely eliminate publication bias. [78]

**Fig 5. Results of a Meta-Analysis of the Effects of Asynchronous Training on the Western Aphasia Battery-Aphasia Quotient**

**Fig6. Results of a Meta-Analysis of the Effects of Asynchronous Training on the Western Aphasia Battery Subscales, Western Aphasia Battery Revised Subscales**
