# Supplementary material for: Current status of Tele-speech language therapy by type and support for patients with post-stroke aphasia: A scoping review
Source: PLoS One. 2025 Mar 25;20(3):e0319805. doi: 10.1371/journal.pone.0319805 (PMC11936174; doi:10.1371/journal.pone.0319805)
Supplement: Attachment — Submitted filename: Response to Reviewers.docx [file pone.0319805.s005.docx]

Reviewer 1

*1. in review papers, it is common to mention the reason(s) why the criteria regarding the study quality for selection of papers are the way they are. It is recommended to mention on what basis such criteria are adopted as the generalizations of findings are under question.*

Response to the reviewer: Thank you for your valuable feedback regarding our research quality selection criteria. We recognize that the rationale for the selection criteria described in the Methods section requires further clarification. To address this, we have revised the "Selection Criteria" subsection in the Methods section (Pages 6–7: Lines 105–112).

“**Selection criteria**

We included studies that investigated the assessment or training methods of Tele-SLT for PWA following a stroke. Studies were excluded if they: 1) involved participants under 18 years of age; 2) combined Tele-SLT with in-person SLT; or 3) were review articles (e.g., systematic reviews, scoping reviews, narrative reviews), case reports, qualitative studies, cost-effectiveness analyses, books, conference proceedings, or study protocols. Full details of the inclusion and exclusion criteria are provided in Table 1.”

*2. it is suggested to include more articles as the number of articles (i.e., 15 articles) may not provide a comprehensive picture of what is going on in that area. So, if it is possible, widen the scope of this scoping review. As it was mentioned in the manuscript, out of 1,484, only 15 met our strict inclusion criteria.*

Response to the reviewer: Thank you for your insightful comments regarding the comprehensiveness of the review. In response, we have revised the study selection criteria to encompass a broader range of research trends. The following changes were made:

1. We removed the restriction on the minimum sample size (n = 20) and reevaluated the selection criteria. This adjustment allowed the inclusion of smaller-scale studies that were previously excluded. Consequently, 20 additional studies were incorporated, increasing the total from 15 to 35—a 133% increase. These articles are presented in Table 1 (See “Table 1. Articles Added This Time” below).
2. Despite expanding the scope of the analysis, as detailed in the Methods (Pages 6–10, Lines 89–163) and Results (Pages 10–31, Lines 165–281), the key research trends in tele-rehabilitation for speech-language therapy remain consistent. This consistency further reinforces the reliability of our findings.

Table1. Articles added this time

| No. | Article |
| --- | --- |
| 1 | Cherney LR, et al. Complexity and Feedback During Script Training in Aphasia: A Feasibility Study. Arch Phys Med Rehabil. 2022;103: 205-214. |
| 2 | Braley M, et al. A Virtual, Randomized, Control Trial of a Digital Therapeutic for Speech, Language, and Cognitive Intervention in Post-stroke Persons With Aphasia. Front Neurol. 2021;12:626780. |
| 3 | Grechuta K, et al. Multisensory Cueing Facilitates Naming in Aphasia. J NeuroEng Rehabil. 2020;17:122. |
| 4 | Øra HP, et al. The Effect of Augmented Speech-Language Therapy Delivered by Telerehabilitation on Poststroke Aphasia—A Pilot Randomized Controlled Trial. Clin Rehabil. 2020; 34: 918-930. |
| 5 | Pitt R, et al. The Impact of the Telerehabilitation Group Aphasia Intervention and Networking Programme on Communication, Participation, and Quality of Life in People with Aphasia. Int J Speech Lang Pathol. 2018; 20: 1-11. |
| 6 | Palmer R, et al. Self-managed, Computerised Speech and Language Therapy for Patients with Chronic Aphasia Post-Stroke Compared with Usual Care or Attention Control (Big CACTUS): A Multicentre, Single-Blinded, Randomised Controlled Trial. Lancet Neurol. 2019; 18: 821-833. |
| 7 | Grechuta K, et al. Augmented Dyadic Therapy Boosts Recovery of Language Function in Patients With Nonfluent Aphasia: A Randomized Controlled Trial. Stroke. 2019; 50: 1270-1274. |
| 8 | Pitt R, et al. The development and feasibility of an online aphasia group intervention and networking program – TeleGAIN. Int J Speech Lang Pathol. 2019; 21: 23-36. |
| 9 | Zhou Q, et al. Telerehabilitation Combined Speech-Language and Cognitive Training Effectively Promoted Recovery in Aphasia Patients. Front Psychol. 2018; 9: 2312. |
| 10 | Pitt R, et al. The Feasibility of Delivering Constraint-Induced Language Therapy via the Internet. Digit Health. 2017; 3: 2055207617718767 |
| 11 | Choi YH, et al. A Telerehabilitation Approach for Chronic Aphasia Following Stroke. Telemed J E Health. 2016; 22: 434-440. |
| 12 | Cherney LR, et al. Impact of Personal Relevance on Acquisition and Generalization of Script Training for Aphasia: A Preliminary Analysis. Am J Speech Lang Pathol. 2015;24: 913-922. |
| 13 | Kurland J, et al. iPractice: Piloting the Effectiveness of a Tablet-Based Home Practice Program in Aphasia Treatment. Semin Speech Lang. 2014; 35: 51-64. |
| 14 | Agostini M, et al. Telerehabilitation in Poststroke Anomia. BioMed Res Int. 2014; 2014:706909. |
| 15 | Palmer R, et al. Computer Therapy Compared With Usual Care for People With Long-Standing Aphasia Poststroke: A Pilot Randomized Controlled Trial. Stroke. 2012; 43: 1904-1911. |
| 16 | Archibald LM, et al. Implementation of Computer-Based Language Therapy in Aphasia. Ther Adv Neurol Disord. 2009; 2: 299-311. |
| 17 | Cherney LR, et al. Novel Technology for Treating Individuals with Aphasia and Concomitant Cognitive Deficits. Top Stroke Rehabil. 2008; 15: 542–554. |
| 18 | Altaib MK, et al. A Feasibility Study of Teleassessment for People with Aphasia in Saudi Arabia and Kuwait. Aphasiology. 2023; 37: 1198–1222. |
| 19 | Furnas DW, et al. The Effect of Computerised Verb Network Strengthening Treatment on Lexical Retrieval in Aphasia. Aphasiology. 2014; 28: 401–420. |
| 20 | van Vuuren S, et al. A Virtual Therapist for Speech and Language Therapy. Intell Virtual Agents. 2014; 8637:438-448. |

*3. because of the low number of the studies, in the discussion section, biases in the number of studies are explained on and because of the strict criteria, the discussion cannot be convincing.
the method section was rigorous as the the degree of agreement was mentioned and coding seems to have been done correctly. In the method, only the inclusion criteria is recommended to change, if possible.*

Response to the reviewer: Thank you for your thoughtful evaluation of the rigor of our methodology. We agree with your observation that while the reliability of the methodology was ensured through stringent measures, the strict inclusion criteria may have narrowed the scope of the analysis and reduced the persuasiveness of the discussion. To address this issue, we have made the following improvements:

1. As detailed in the Methods section (Pages 6–10, Lines 89–163), we removed the sample size criterion (n ≥ 20) and included smaller-scale studies in our analysis. This revision increased the number of studies from 15 to 35 (Fig 1, Page 11).
2. In the Results section (Pages 10–31, Lines 165–280), we have added a detailed description of the findings based on the expanded dataset.
3. In the Discussion section (Pages 32–38, Lines 291–389), we broadened our interpretation to reflect the expanded evidence base and provided a more thorough examination of the generalizability of the findings.

Additionally, we have preserved the rigor of other methodological aspects, such as the reliability of the coding process, in accordance with established procedures.

Fig 1. PRISMA Flow Diagram Illustrating the Selection of Articles Included in the Scoping Review.

Reviewer 2

*Although the topic [Current status of tele-speech language therapy by type and support for patients with post-stroke aphasia: a scoping review] is quite interesting, the article is only a review paper offering very limited insights which I believe are not significant enough to be considered for publication at a journal like PlosOne. The authors could perhaps consider either adding experimental data / case studies, or submitting the paper to a lower level journal.*

Response to the reviewer: Thank you for your thoughtful feedback. We have addressed your concerns by expanding our study scope and conducting a comprehensive additional literature review. As a result, 20 new studies were included, increasing the total to 35. We also performed an exploratory meta-analysis on the training effects of telepractice speech-language therapy (Tele-SLT). The 20 additional papers are listed in Table 1 (See “Table 1. Articles Added This Time” below) and Fig. 1, while the results of the meta-analysis are detailed in the "Training Effects of Tele-SLT" section of the Results (Page 31, Lines 265–280, Figs. 4–6).

Our findings revealed that asynchronous training positively impacted language functions in individuals with aphasia, whereas synchronous training did not show a significant effect on communication skills. Based on these insights, we have significantly enhanced the discussion to provide a more comprehensive analysis.

A scoping review including 35 studies on support for individuals with aphasia is uncommon, even among recent similar studies (e.g., Currie, S. S., et al., 2024; Hernandez, N. J., et al., 2024). Furthermore, meta-analyses specifically focused on Tele-SLT training remain extremely limited. We believe this study offers a valuable contribution by providing a broader and more detailed analysis compared to prior research. We sincerely hope that the revisions meet your expectations.

Table 1. Articles added this time

| No. | Article |
| --- | --- |
| 1 | Cherney LR, et al. Complexity and Feedback During Script Training in Aphasia: A Feasibility Study. Arch Phys Med Rehabil. 2022;103: 205-214. |
| 2 | Braley M, et al. A Virtual, Randomized, Control Trial of a Digital Therapeutic for Speech, Language, and Cognitive Intervention in Post-stroke Persons with Aphasia. Front Neurol. 2021; 12:626780. |
| 3 | Grechuta K, et al. Multisensory Cueing Facilitates Naming in Aphasia. J NeuroEng Rehabil. 2020; 17:122. |
| 4 | Øra HP, et al. The Effect of Augmented Speech-Language Therapy Delivered by Telerehabilitation on Poststroke Aphasia—A Pilot Randomized Controlled Trial. Clin Rehabil. 2020; 34: 918-930. |
| 5 | Pitt R, et al. The Impact of the Telerehabilitation Group Aphasia Intervention and Networking Programme on Communication, Participation, and Quality of Life in People with Aphasia. Int J Speech Lang Pathol. 2018; 20: 1-11. |
| 6 | Palmer R, et al. Self-managed, Computerised Speech and Language Therapy for Patients with Chronic Aphasia Post-Stroke Compared with Usual Care or Attention Control (Big CACTUS): A Multicentre, Single-Blinded, Randomised Controlled Trial. Lancet Neurol. 2019; 18: 821-833. |
| 7 | Grechuta K, et al. Augmented Dyadic Therapy Boosts Recovery of Language Function in Patients With Nonfluent Aphasia: A Randomized Controlled Trial. Stroke. 2019; 50: 1270-1274. |
| 8 | Pitt R, et al. The development and feasibility of an online aphasia group intervention and networking program – TeleGAIN. Int J Speech Lang Pathol. 2019; 21: 23-36. |
| 9 | Zhou Q, et al. Telerehabilitation Combined Speech-Language and Cognitive Training Effectively Promoted Recovery in Aphasia Patients. Front Psychol. 2018; 9: 2312. |
| 10 | Pitt R, et al. The Feasibility of Delivering Constraint-Induced Language Therapy via the Internet. Digit Health. 2017; 3: 2055207617718767. |
| 11 | Choi YH, et al. A Telerehabilitation Approach for Chronic Aphasia Following Stroke. Telemed J E Health. 2016; 22: 434-440. |
| 12 | Cherney LR, et al. Impact of Personal Relevance on Acquisition and Generalization of Script Training for Aphasia: A Preliminary Analysis. Am J Speech Lang Pathol. 2015;24: 913-922. |
| 13 | Kurland J, et al. iPractice: Piloting the Effectiveness of a Tablet-Based Home Practice Program in Aphasia Treatment. Semin Speech Lang. 2014; 35: 51-64. |
| 14 | Agostini M, et al. Telerehabilitation in Poststroke Anomia. BioMed Res Int. 2014; 2014:706909. |
| 15 | Palmer R, et al. Computer Therapy Compared with Usual Care for People With Long-Standing Aphasia Poststroke: A Pilot Randomized Controlled Trial. Stroke. 2012; 43: 1904-1911. |
| 16 | Archibald LM, et al. Implementation of Computer-Based Language Therapy in Aphasia. Ther Adv Neurol Disord. 2009; 2: 299-311. |
| 17 | Cherney LR, et al. Novel Technology for Treating Individuals with Aphasia and Concomitant Cognitive Deficits. Top Stroke Rehabil. 2008; 15: 542–554. |
| 18 | Altaib MK, et al. A Feasibility Study of Teleassessment for People with Aphasia in Saudi Arabia and Kuwait. Aphasiology. 2023; 37: 1198–1222. |
| 19 | Furnas DW, et al. The Effect of Computerised Verb Network Strengthening Treatment on Lexical Retrieval in Aphasia. Aphasiology. 2014; 28: 401–420. |
| 20 | van Vuuren S, et al. A Virtual Therapist for Speech and Language Therapy. Intell Virtual Agents. 2014; 8637:438-448. |

Fig 1. PRISMA Flow Diagram Illustrating the Selection of Articles Included in the Scoping Review.

**“Training effects of Tele-SLT (Page 31, Lines 265–280)**

We performed a meta-analysis of Tele-SLT training effects using data from four RCTs. Two studies examined synchronous training using the CAL and CETI as outcome measures [38, 41], and two examined asynchronous training using the WAB as the outcome measure [44, 55]. For synchronous training, there was no significant difference between the intervention and control groups in combined CAL and CETI scores (fixed-effects model, SMD = -0.10, 95% CI = -0.57 to 0.37, *P* > 0.05) (Figure 4). For asynchronous training, analysis of the WAB-AQ showed no significant difference between groups (fixed-effects model, MD = 8.55, 95% CI = -4.59 to 21.70, *P* > 0.05) (Figure 5). However, a significant difference was found in the combined WAB subscales (Comprehension, Naming, Repetition) (fixed-effects model, MD = 0.70, 95% CI = 0.03 to 1.37, *P* < 0.05). Despite the significant result for the combined subscales, no significant differences were observed for individual subscales: Comprehension (fixed-effects model, MD = 0.46, 95% CI = -0.42 to 1.34, *P* > 0.05), Naming (fixed-effects model, MD = 1.21, 95% CI = -0.23 to 2.65, *P* > 0.05), and Repetition (fixed-effects model, MD = 0.83, 95% CI = -0.61 to 2.28, *P* > 0.05) (Fig 6)”

Fig. 4. Results of a Meta-Analysis of the Effects of Synchronous Training on Communication Outcomes

Fig 5. Results of a Meta-Analysis of the Effects of Asynchronous Training on the Western Aphasia Battery-Aphasia Quotient

Fig 6. Results of a Meta-Analysis of the Effects of the Western Aphasia Battery Subscales on Asynchronous Training

Reviewer 3

1. *Limited to English and Japanese language studies, potentially missing relevant research in other languages*

Response to the reviewer: Thank you for raising these important points.

In this study, we followed the methodology of prior reviews in the field of aphasia (e.g., Wang, G., et al., 2020) and limited our scope to studies written in the authors' native language, Japanese, and in English, which encompass a significant portion of relevant research. However, as you rightly pointed out, excluding studies published in other languages may have introduced selection bias.

Although directly addressing this issue within the scope of this study is challenging, we have acknowledged it as a limitation in the Limitations section (Page 37, Lines 383–385).

“Second, while we performed a comprehensive literature search, the exclusion of non-English and non-Japanese articles and the absence of hand-searching of grey literature or specific databases may have introduced selection bias.”

2. *The methods section is generally well-described. The use of the PRISMA-ScR guidelines is appropriate. However, the authors could provide more detail on how disagreements in the screening process were resolved.*

Response to the reviewer: Thank you very much for your valuable feedback. We appreciate your recognition of the appropriateness of the Methods section and the application of the PRISMA-ScR guidelines.

As you correctly pointed out, our original description lacked sufficient detail on how disagreements during the screening process were resolved. To address this, we have revised the "Screening Method" and "Data Extraction Process" subsections in the Methods section to provide a more detailed explanation (Pages 8–9, Lines 115–136).

“**Screening method**

Two authors (YK and SN) independently screened titles and abstracts of identified articles. Subsequently, two other authors (YK and AY) independently reviewed the full texts of the selected articles for inclusion. Any discrepancies between authors during the screening process were resolved through discussion. If a consensus could not be reached, a third author (HN) made the final decision.

**Data extraction process**

Two authors (YK and AY) independently reviewed the full texts of articles selected in the second screening and extracted data. Discrepancies in data extraction were resolved through discussion between the two authors. If consensus could not be reached, a third author (HN) made the final decision. For studies on tele-assessment methods, the following information was collected: author, year of publication, country of the first author, participant characteristics (e.g., gender, age), number of participants, domains covered (e.g., language function, communication, well-being, QOL), software used, electronic devices used, and scale accuracy (e.g., reliability, validity). For studies on tele-training methods, the following information was collected: author, year of publication, study design, participant characteristics (gender, age), number of participants, domains covered (language function, communication, well-being, QOL), software used, electronic devices used, and training outcomes. One author (YK) managed the data using Rayyan reference management software and compiled the data into tables using Excel. Any missing articles were obtained by contacting the corresponding authors.”

3. *Consider including a flow diagram of the study selection process (PRISMA flow diagram)*

Response to the reviewer: Thank you very much for your valuable suggestion.

We agree that including a PRISMA flow diagram is essential for clearly presenting the study selection process. In response, we have incorporated a PRISMA flow diagram into the manuscript (Fig. 1, Page 11), which visually depicts the literature search and selection process. We believe this addition significantly enhances the clarity, transparency, and reproducibility of our study.

Once again, we sincerely appreciate your insightful advice.

Fig 1. PRISMA Flow Diagram Illustrating the Selection of Articles Included in the Scoping Review.

4. *Provide more details on the data extraction process and how data were synthesized*

Response to the reviewer: We sincerely thank you for your valuable feedback.

We agree that our explanation of the data extraction process and data integration methods was insufficient for readers. In response to your suggestion, we have added further details to the "Data Extraction Process" subsection in the Methods section and revised it accordingly (Pages 8–9, Lines 121–136). Additionally, for the 90 papers subjected to secondary screening, both the evaluator's ratings and the final ratings are now presented in Table S3.

Once again, we greatly appreciate your insightful suggestions, which have helped us improve the clarity and transparency of our study.

“**Data extraction process**

Two authors (YK and AY) independently reviewed the full texts of articles selected in the second screening and extracted data. Discrepancies in data extraction were resolved through discussion between the two authors. If consensus could not be reached, a third author (HN) made the final decision. For studies on tele-assessment methods, the following information was collected: author, year of publication, country of the first author, participant characteristics (e.g., gender, age), number of participants, domains covered (e.g., language function, communication, well-being, QOL), software used, electronic devices used, and scale accuracy (e.g., reliability, validity). For studies on tele-training methods, the following information was collected: author, year of publication, study design, participant characteristics (gender, age), number of participants, domains covered (language function, communication, well-being, QOL), software used, electronic devices used, and training outcomes. One author (YK) managed the data using Rayyan reference management software and compiled the data into tables using Excel. Any missing articles were obtained by contacting the corresponding authors.”

Table S3. Results by evaluator and final evaluation of the secondary screening inclusion articles

| No. | Secondary screening inclusion articles | Evaluator 1 (YK) | Evaluator 2 (AY) | Final decision | Reasons for the decision to exclude |
| --- | --- | --- | --- | --- | --- |
| 1 | Trebilcock M, et al. Pilot trial of the online implementation intervention Aphasia Nexus: Connecting Evidence to Practice. Int J Speech Lang Pathol. 2024; 26: 16-27. (Published online: 23 Dec 2022) | Exclude | Exclude | Exclude | Different study designs |
| 2 | de Grosbois J, et al. Asynchronous, online spaced-repetition training alleviates word-finding difficulties in aphasia. Neuropsychol Rehabil. 2023; 10: 1672-1692. (Published online: 15 Nov 2022) | Exclude | Exclude | Exclude | Different target group |
| 3 | Quique YM, et al. Applying adaptive distributed practice to self-managed computer-based anomia treatment: A single-case experimental design. J Commun Disord. 2022; 106249. | Exclude | Exclude | Exclude | Different methods of support and intervention |
| 4 | Liu M, et al. Improvement in language function in patients with aphasia using computer-assisted executive function training: A controlled clinical trial. PM R. 2022; 14: 913-921. | Exclude | Exclude | Exclude | Different methods of support and intervention |
| 5 | Trebilcock M, et al. Development of an online implementation intervention for aphasia clinicians to increase the intensity and comprehensiveness of their service. Disabil Rehabil. 2022; 44: 4629-4638. | Exclude | Exclude | Exclude | Different study designs |
| 6 | Rohde A, et al. Inter-rater reliability, intra-rater reliability and internal consistency of the Brisbane Evidence-Based Language Test. Disabil Rehabil. 2022; 44: 637-645. | Exclude | Exclude | Exclude | Different target group |
| 7 | Spaccavento S, et al. Effects of computer-based therapy versus therapist-mediated therapy in stroke-related aphasia: Pilot non-inferiority study. J Commun Disord. 2021; 94: 106158. | Exclude | Include | Include | - |
| 8 | Jacobs M, et al. Estimating the cost and value of functional changes in communication ability following telepractice treatment for aphasia. PLoS One. 2021; 16: e0257462. | Exclude | Exclude | Exclude | Different target group |
| 9 | Cherney LR, et al. Web-based Oral Reading for Language in Aphasia (Web ORLA(®)): A pilot randomized control trial. Clin Rehabil. 2021; 35: 976-987. | Include | Include | Include | - |
| 10 | Harrison M, et al. Factors Associated With Adherence to Self-Managed Aphasia Therapy Practice on a Computer-A Mixed Methods Study Alongside a Randomized Controlled Trial. Front Neurol. 2020; 11: 582328. | Exclude | Exclude | Exclude | Different study designs |
| 11 | Giachero A, et al. Conversational Therapy through Semi-Immersive Virtual Reality Environments for Language Recovery and Psychological Well-Being in Post Stroke Aphasia. Behav Neurol. 2020: 2846046. | Include | Include | Include | - |
| 12 | Dekhtyar M, et al. Videoconference Administration of the Western Aphasia Battery–Revised: Feasibility and Validity. Am J Speech Lang Pathol. 2020; 29: 673-687. | Exclude | Exclude | Exclude | Different target group |
| 13 | Cherney LR, et al. Optimising recovery in aphasia: Learning following exposure to a single dose of computer-based script training. Int J Speech Lang Pathol. 2019; 21: 448-458. | Include | Include | Include | - |
| 14 | Maresca G, et al. Toward Improving Poststroke Aphasia: A Pilot Study on the Growing Use of Telerehabilitation for the Continuity of Care. J Stroke Cerebrovasc Dis. 2019; 28: 104303. | Include | Include | Include | - |
| 15 | Kerry SJ, et al. How Does iReadMore Therapy Change the Reading Network of Patients with Central Alexia? J Neurosci. 2019; 39: 5719-5727. | Exclude | Exclude | Exclude | Different target group |
| 16 | Marshall J, et al. Technology-enhanced writing therapy for people with aphasia: results of a quasi-randomized waitlist controlled study. Int J Lang Commun Disord. 2019; 54: 203-220. | Exclude | Exclude | Exclude | Different methods of support and intervention |
| 17 | Klischies D, et al. Evaluation of Deep Clustering for Diarization of Aphasic Speech. Stud Health Technol Inform. 2019; 260: 81-88. | Exclude | Exclude | Exclude | Different publication classifications |
| 18 | Wall KJ, et al. Using technology to overcome the language barrier: the Cognitive Assessment for Aphasia App. Disabil Rehabil. 2018; 40: 1333-1344. | Exclude | Exclude | Exclude | Different study designs |
| 19 | Kurland J, et al. Effects of a Tablet-Based Home Practice Program With Telepractice on Treatment Outcomes in Chronic Aphasia. J Speech Lang Hear Res. 2018; 61: 1140-1156. | Include | Include | Include | - |
| 20 | Kesav P, et al. Effectiveness of speech language therapy either alone or with add-on computer-based language therapy software (Malayalam version) for early post stroke aphasia: A feasibility study. J Neurol Sci. 2017; 380: 137-141. | Exclude | Exclude | Exclude | Different methods of support and intervention |
| 21 | Macoir J, et al. In-Home Synchronous Telespeech Therapy to Improve Functional Communication in Chronic Poststroke Aphasia: Results from a Quasi-Experimental Study. Telemed J E Health. 2017; 23: 630-639. | Include | Include | Include | - |
| 22 | Roper A, et al. Benefits and Limitations of Computer Gesture Therapy for the Rehabilitation of Severe Aphasia. Front Hum Neurosci. 2016; 10: 595. | Exclude | Exclude | Exclude | Different methods of support and intervention |
| 23 | Marshall J, et a. Evaluating the Benefits of Aphasia Intervention Delivered in Virtual Reality: Results of a Quasi-Randomised Study. PLoS One. 2016; 11: e0160381. | Include | Include | Include | - |
| 24 | Ruiter MB, et al. An Exploratory Investigation of E-Rest: Teletherapy for Chronically Aphasic Speakers. Int J Telerehabil. 2016; 8: 21-28. | Exclude | Exclude | Exclude | Different study designs |
| 25 | Woolf C, et al. A comparison of remote therapy, face to face therapy and an attention control intervention for people with aphasia: a quasi-randomised controlled feasibility study. Clin Rehabil. 2016; 30: 359-373. | Exclude | Exclude | Exclude | Different methods of support and intervention |
| 26 | Varley R, et al. Self-Administered Computer Therapy for Apraxia of Speech: Two-Period Randomized Control Trial With Crossover. Stroke. 2016; 47: 822-828. | Include | Exclude | Exclude | Different target group |
| 27 | van Vuuren S, et al. A Virtual Therapist for Speech and Language Therapy. Intell Virtual Agents. 2014; 8637:438-448. | Include | Include | Include | - |
| 28 | Latimer NR, et al. Cost-utility of self-managed computer therapy for people with aphasia. Int J Technol Assess Health Care. 2013; 29 :402-409. | Exclude | Exclude | Exclude | Different study designs |
| 29 | Hussmann K, et al. Computer-assisted analysis of spontaneous speech: quantification of basic parameters in aphasic and unimpaired language. Clin Linguist Phon. 2012; 26: 661-680. | Exclude | Exclude | Exclude | Different target group |
| 30 | Cherney LR, et al. Computer-based script training for aphasia: emerging themes from post-treatment interviews. J Commun Disord. 2011; 44: 493-501. | Exclude | Exclude | Exclude | Different study designs |
| 31 | Cherney LR. Oral reading for language in aphasia (ORLA): evaluating the efficacy of computer-delivered therapy in chronic nonfluent aphasia. Top Stroke Rehabil. 2010; 17: 423-431. | Include | Include | Include | - |
| 32 | Manheim LM, et al. Patient-reported changes in communication after computer-based script training for aphasia. Arch Phys Med Rehabil. 2009; 90: 623-627. | Include | Include | Include | - |
| 33 | Palsbo SE. Equivalence of functional communication assessment in speech pathology using videoconferencing. J Telemed Telecare. 2007; 13: 40-43. | Exclude | Include | Exclude | Different target group |
| 34 | Laganaro M., et al. Acquired alexia in multilingual aphasia and computer-assisted treatment in both languages: issues of generalisation and transfer. Folia Phoniatr Logop. 2002; 53: 135-144. | Exclude | Exclude | Exclude | Different study designs |
| 35 | Aftonomos LB, et al. Improving outcomes for persons with aphasia in advanced community-based treatment programs. Stroke. 1999; 30: 1370-1379. | Exclude | Exclude | Exclude | Different target group |
| 36 | Katz RC, et al. The efficacy of computer-provided reading treatment for chronic aphasic adults. J Speech Lang Hear Res. 1997; 40: 493-507. | Include | Include | Include | - |
| 37 | De Luca R, et al. Virtual reality as a new tool for the rehabilitation of post-stroke patients with chronic aphasia: an exploratory study. Aphasiology. 2023; 37: 249-259. | Exclude | Exclude | Exclude | Different methods of support and intervention |
| 38 | Macedonia M, et al. Gesture based word (re)acquisition with a virtual agent in augmented reality: A preliminary study. Annu Rev CyberTher Telemed. 2022; 20: 83-86. | Exclude | Exclude | Exclude | Different target group |
| 39 | Cruice M, et al. Preliminary outcomes from a pilot study of personalised online supported conversation for participation intervention for people with Aphasia. Aphasiology. 2021; 35: 1293-1317. | Include | Include | Include | - |
| 40 | Grasso SM, et al. A tablet-based home practice program paired with telepractice promotes maintenance and learning of objects and actions in individuals with chronic aphasia. Evid Base Commun Assess Interv. 2019; 13: 171-176. | Exclude | Exclude | Exclude | Different target group |
| 41 | Amaya A, et al. Receiving aphasia intervention in a virtual environment: the participants’ perspective. Aphasiology. 2018; 32: 538-558. | Include | Include | Include | - |
| 42 | Hess DC, et al. Stroke telepresence: Removing all geographic barriers. Neurology. 2011; 76: 1121-1123. | Exclude | Exclude | Exclude | Different publication classifications |
| 43 | Steele RD, et al. Outcome improvements in persons with chronic global aphasia following the use of a speech-generating device. Acta Neuropsychologica. 2010; 8: 342-359. | Exclude | Exclude | Exclude | Different target group |
| 44 | Hill MAJ, et al. The effects of aphasia severity on the ability to assess language disorders via telerehabilitation. Aphasiology. 2009; 23: 627-642. | Exclude | Exclude | Exclude | Different target group |
| 45 | Bruckert R, et al. The use of a computer driven videodisc for the assessment and rehabilitation of aphasia. Aphasiology. 1989; 3: 473-478. | Exclude | Exclude | Exclude | Different publication classifications |
| 46 | De Luca R, et al. Computerized Training in Poststroke Aphasia: what About the Long-Term Effects? A Randomized Clinical Trial. J Stroke Cerebrovasc Dis. 2018; 27: 2271-2276. | Exclude | Exclude | Exclude | Different target group |
| 47 | Automatic Assessment of Aphasic Speech Sensed by Audio Sensors for Classification into Aphasia Severity Levels to Recommend Speech Therapies. | Exclude | Exclude | Exclude | Different target group |
| 48 | Guo YE, et al. Assessment of Aphasia Across the International Classification of Functioning, Disability and Health Using an iPad-Based Application. Telemed J E Health. 2017; 23: 313-326. | Include | Include | Include | - |
| 49 | Choi YH, et al. A Telescreening Tool to Detect Aphasia in Patients with Stroke. Telemed J E Health. 2015; 21: 729-734. | Include | Exclude | Include | - |
| 50 | Cherney LR, et al. Complexity and Feedback During Script Training in Aphasia: A Feasibility Study. Arch Phys Med Rehabil. 2022;103: 205-214. | Include | Include | Include | - |
| 51 | Musso M, et al. Aphasia Recovery by Language Training Using a Brain–Computer Interface: A Proof-of-Concept Study. Brain Commun. 2022; 4: fcac008. | Exclude | Exclude | Exclude | Different methods of support and intervention |
| 52 | Velez JA, et al. A Visual Interactive Narrative Intervention (VINI) for Aphasia Education: Can Digital Applications Administer Augmented Input to Educate Stroke Survivors with Aphasia? Patient Educ Couns. 2021; 104: 2536-2543. | Exclude | Exclude | Exclude | Different methods of support and intervention |
| 53 | Braley M, et al. A Virtual, Randomized, Control Trial of a Digital Therapeutic for Speech, Language, and Cognitive Intervention in Post-stroke Persons With Aphasia. Front Neurol. 2021;12:626780. | Include | Include | Include | - |
| 54 | Gallée J, et al. The Application of Lexical Retrieval Training in Tablet-Based Speech-Language Intervention. Front Neurol. 2020;11:583246. | Exclude | Exclude | Exclude | Different target group |
| 55 | Grechuta K, et al. Multisensory Cueing Facilitates Naming in Aphasia. J NeuroEng Rehabil. 2020;17:122. | Include | Include | Include | - |
| 56 | Øra HP, et al. The Effect of Augmented Speech-Language Therapy Delivered by Telerehabilitation on Poststroke Aphasia—A Pilot Randomized Controlled Trial. Clin Rehabil. 2020; 34: 918-930. | Include | Include | Include | - |
| 57 | Pitt R, et al. The Impact of the Telerehabilitation Group Aphasia Intervention and Networking Programme on Communication, Participation, and Quality of Life in People with Aphasia. Int J Speech Lang Pathol. 2018; 20: 1-11. | Include | Include | Include | - |
| 58 | Palmer R, et al. Self-managed, Computerised Speech and Language Therapy for Patients with Chronic Aphasia Post-Stroke Compared with Usual Care or Attention Control (Big CACTUS): A Multicentre, Single-Blinded, Randomised Controlled Trial. Lancet Neurol. 2019; 18: 821-833. | Include | Include | Include | - |
| 59 | Grechuta K, et al. Augmented Dyadic Therapy Boosts Recovery of Language Function in Patients With Nonfluent Aphasia: A Randomized Controlled Trial. Stroke. 2019; 50: 1270-1274. | Include | Include | Include | - |
| 60 | Pitt R, et al. The development and feasibility of an online aphasia group intervention and networking program – TeleGAIN. Int J Speech Lang Pathol. 2019; 21: 23-36. | Include | Include | Include | - |
| 61 | Rhodes NC, et al. Script Training Using Telepractice With Two Adults With Chronic Non-Fluent Aphasia. Int J Telerehabil. 2018; 10: 89-104. | Exclude | Exclude | Exclude | Different target group |
| 62 | Zhou Q, et al. Telerehabilitation Combined Speech-Language and Cognitive Training Effectively Promoted Recovery in Aphasia Patients. Front Psychol. 2018; 9: 2312. | Include | Include | Include | - |
| 63 | Pitt R, et al. The Feasibility of Delivering Constraint-Induced Language Therapy via the Internet. Digit Health. 2017; 3: 2055207617718767 | Include | Include | Include | - |
| 64 | Thiel L, et al. Promoting Linguistic Complexity, Greater Message Length, and Ease of Engagement in Email Writing in People with Aphasia: Initial Evidence from a Study Utilizing Assistive Writing Software. Int J Lang Commun Disord. 2016; 00: 1-19. | Exclude | Exclude | Exclude | Different methods of support and intervention |
| 65 | Hill AJ, et al. Refining an Asynchronous Telerehabilitation Platform for Speech-Language Pathology: Engaging End-Users in the Process. Front Hum Neurosci. 2016; 10: 640. | Exclude | Exclude | Exclude | Different study designs |
| 66 | Best W, et al. Conversation Therapy with People with Aphasia and Conversation Partners Using Video Feedback: A Group and Case Series Investigation of Changes in Interaction. Front Hum Neurosci. 2016; 10:562. | Exclude | Exclude | Exclude | Different methods of support and intervention |
| 67 | Simic T, et al. A Usability Study of Internet-Based Therapy for Naming Deficits in Aphasia. Am J Speech Lang Pathol. 2016; 25: 581-593. | Exclude | Exclude | Exclude | Different study designs |
| 68 | Choi YH, et al. A Telerehabilitation Approach for Chronic Aphasia Following Stroke. Telemed J E Health. 2016; 22: 434-440. | Include | Include | Include | - |
| 69 | Cherney LR, et al. Impact of Personal Relevance on Acquisition and Generalization of Script Training for Aphasia: A Preliminary Analysis. Am J Speech Lang Pathol. 2015;24: 913-922. | Include | Include | Include | - |
| 70 | Steele RD, et al. Combining Teletherapy and On-Line Language Exercises in the Treatment of Chronic Aphasia: An Outcome Study. Int J Telerehabil. 2014;6(2):3-1. | Exclude | Exclude | Exclude | Different methods of support and intervention |
| 71 | Kurland J, et al. iPractice: Piloting the Effectiveness of a Tablet-Based Home Practice Program in Aphasia Treatment. Semin Speech Lang. 2014; 35: 51-64. | Include | Include | Include | - |
| 72 | Agostini M, et al. Telerehabilitation in Poststroke Anomia. BioMed Res Int. 2014; 2014:706909. | Include | Include | Include | - |
| 73 | Goldberg S, et al. Script Training and Generalization for People With Aphasia. Am J Speech Lang Pathol. 2012; 21: 222-238. | Exclude | Exclude | Exclude | Different target group |
| 74 | Palmer R, et al. Computer Therapy Compared With Usual Care for People With Long-Standing Aphasia Poststroke: A Pilot Randomized Controlled Trial. Stroke. 2012; 43: 1904-1911. | Include | Include | Include | - |
| 75 | Dechêne L, et al. Simulated In-Home Teletreatment for Anomia: A Pilot Study. Int J Telerehabil. 2011; 3: 3-10. | Exclude | Exclude | Exclude | Different methods of support and intervention |
| 76 | Adrián JA, et al. Extending the Use of Spanish Computer-Assisted Anomia Rehabilitation Program (CARP-2) in People with Aphasia. J Commun Disord. 2011; 44: 666-677. | Exclude | Exclude | Exclude | Different target group |
| 77 | Nicholas ML, et al. C-Speak Aphasia Alternative Communication Program for People with Severe Aphasia: Importance of Executive Functioning and Semantic Knowledge. Neuropsychol Rehabil. 2011; 21: 322-366. | Exclude | Exclude | Exclude | Different methods of support and intervention |
| 78 | Thompson CK, et al. Sentactics®: Computer-Automated Treatment of Underlying Forms in Agrammatic Aphasia. Aphasiology. 2010; 24: 1242-1266. | Exclude | Exclude | Exclude | Different target group |
| 79 | Archibald LM, et al. Implementation of Computer-Based Language Therapy in Aphasia. Ther Adv Neurol Disord. 2009; 2: 299-311. | Include | Include | Include | - |
| 80 | Cherney LR, et al. Novel Technology for Treating Individuals with Aphasia and Concomitant Cognitive Deficits. Top Stroke Rehabil. 2008; 15: 542–554. | Include | Include | Include | - |
| 81 | Laganaro M, et al. Computerised Treatment of Anomia in Acute Aphasia: Treatment Intensity and Training Size. Neuropsychol Rehabil. 2006; 16: 630-640. | Exclude | Exclude | Exclude | Different target group |
| 82 | Van de Sandt-Koenderman M, et al. A Computerised Communication Aid for People with Aphasia. Disabil Rehabil. 2005; 27: 529–533. | Exclude | Exclude | Exclude | Different target group |
| 83 | Altaib MK, et al. A Feasibility Study of Teleassessment for People with Aphasia in Saudi Arabia and Kuwait. Aphasiology. 2023; 37: 1198–1222. | Include | Include | Include | - |
| 84 | Zannino GD, et al. Picture and Spoken Word Presentation in Repetition Training for Anomia: Does Stimulus Order Matter? Evidence from a Computer-Based Telemedicine Approach. Aphasiology. 2020; 34: 275-299. | Exclude | Exclude | Exclude | Different target group |
| 85 | Furnas DW, et al. The Effect of Computerised Verb Network Strengthening Treatment on Lexical Retrieval in Aphasia. Aphasiology. 2014; 28: 401–420. | Include | Include | Include | - |
| 86 | Pedersen PM, et al. Improvement of Oral Naming by Unsupervised Computerised Rehabilitation. Aphasiology. 2001; 15: 151-169. | Exclude | Exclude | Exclude | Different study designs |
| 87 | Duffy JR, et al. Telemedicine and the Diagnosis of Speech and Language Disorders. Mayo Clin Proc. 1997; 72: 1116-1122. | Exclude | Exclude | Exclude | Different target group |
| 88 | Bartlett MR, et al. Informativeness Ratings of Messages Created on an AAC Processing Prosthesis. Aphasiology. 2007; 21: 475-498. | Exclude | Exclude | Exclude | Different methods of support and intervention |
| 89 | Wade J, et al. Talk About IT: Views of People With Aphasia and Their Partners on Receiving Remotely Monitored Computer-Based Word Finding Therapy. Aphasiology. 2003; 17: 1031-1056. | Exclude | Exclude | Exclude | Different study designs |
| 90 | Deloche G, et al. Confrontation Naming Rehabilitation in Aphasics: A Computerised Written Technique. Neuropsychol Rehabil. 1992; 2: 117-124. | Exclude | Exclude | Exclude | Different methods of support and intervention |

5. *Discuss the potential impact of excluding non-English and non-Japanese studies on the review's findings*

Response to the reviewer: Thank you very much for your valuable feedback.

As you rightly pointed out, our discussion did not sufficiently address the potential impact of excluding studies published in languages other than English and Japanese on the results of this review. To address this, we have added a detailed discussion of this issue in the Limitations section (Pages 37–38, Lines 380–389).

We sincerely appreciate your insight, which has helped us enhance the depth and transparency of our discussion.

“Second, while we performed a comprehensive literature search, the exclusion of non-English and non-Japanese articles and the absence of hand-searching of grey literature or specific databases may have introduced selection bias.”
